# The MEK1/2-inhibitor ATR-002 efficiently blocks SARS-CoV-2 propagation and alleviates pro-inflammatory cytokine/chemokine responses

**DOI:** 10.1007/s00018-021-04085-1

**Published:** 2022-01-10

**Authors:** André Schreiber, Dorothee Viemann, Jennifer Schöning, Sebastian Schloer, Angeles Mecate Zambrano, Linda Brunotte, Aileen Faist, Michael Schöfbänker, Eike Hrincius, Helen Hoffmann, Markus Hoffmann, Stefan Pöhlmann, Ursula Rescher, Oliver Planz, Stephan Ludwig

**Affiliations:** 1 Institute of Virology (IVM), Centre for Molecular Biology of Inflammation, University of Muenster, Von-Esmarch-Straße 56, 48149 Münster, North Rhine-Westphalia Germany; 2 CiM-IMPRS Graduate School, University of Muenster, 48149 Münster, North Rhine-Westphalia Germany; 3 Translational Pediatrics, Department of Pediatrics, University Hospital Wuerzburg, 97080 Würzburg, Bavaria Germany; 4 Center for Infection Research, University Wuerzburg, 97080 Würzburg, Bavaria Germany; 5 Cluster of Excellence RESIST (EXC 2155, Hannover Medical School, 30625 Hannover, Lower Saxony Germany; 6 Research Group Regulatory Mechanisms of Inflammation, Institute of Medical Biochemistry, Centre for Molecular Biology of Inflammation, University of Muenster, 48149 Münster, North Rhine-Westphalia Germany; 7 Atriva Therapeutics GmbH, 72072 Tübingen, Baden-Württemberg Germany; 8 Department of Immunology, Interfaculty Institute for Cell Biology, Eberhard Karls University, 72074 Tübingen, Baden-Württemberg Germany; 9 Infection Biology Unit, German Primate Center - Leibniz Institute for Primate Research, Göttingen, Germany; 10 Faculty of Biology and Psychology, University Goettingen, 37077 Göttingen, Lower Saxony Germany; 11 Interdisciplinary Center of Clinical Research (IZKF), Medical Faculty, University of Muenster, 48149 Münster, North Rhine-Westphalia Germany

**Keywords:** SARS-CoV-2, COVID-19, Antiviral drug, Raf/MEK/ERK, MEK1/2-inhibitor, ATR-002

## Abstract

Coronavirus disease 2019 (COVID-19), the illness caused by a novel coronavirus now called severe acute respiratory syndrome coronavirus 2 (SARS-CoV-2), has led to more than 260 million confirmed infections and 5 million deaths to date. While vaccination is a powerful tool to control pandemic spread, medication to relieve COVID-19-associated symptoms and alleviate disease progression especially in high-risk patients is still lacking. In this study, we explore the suitability of the rapid accelerated fibrosarcoma/mitogen-activated protein kinase/extracellular signal-regulated kinase (Raf/MEK/ERK) pathway as a druggable target in the treatment of SARS-CoV-2 infections. We find that SARS-CoV-2 transiently activates Raf/MEK/ERK signaling in the very early infection phase and that ERK1/2 knockdown limits virus replication in cell culture models. We demonstrate that ATR-002, a specific inhibitor of the upstream MEK1/2 kinases which is currently evaluated in clinical trials as an anti-influenza drug, displays strong anti-SARS-CoV-2 activity in cell lines as well as in primary air–liquid-interphase epithelial cell (ALI) cultures, with a safe and selective treatment window. We also observe that ATR-002 treatment impairs the SARS-CoV-2-induced expression of pro-inflammatory cytokines, and thus might prevent COVID-19-associated hyperinflammation, a key player in COVID-19 progression. Thus, our data suggest that the Raf/MEK/ERK signaling cascade may represent a target for therapeutic intervention strategies against SARS-CoV-2 infections and that ATR-002 is a promising candidate for further drug evaluation.

**Supplementary Information:**

The online version contains supplementary material available at 10.1007/s00018-021-04085-1.

## Introduction

Since the emergence of the highly pathogenic and transmissible new betacoronavirus SARS-CoV-2 (2019-nCoV) in Wuhan City, China, in December 2019 [1], more than 260 million confirmed cases of SARS-CoV-2-elicited coronavirus disease 2019 (COVID-19) and over 5 million deaths worldwide were recorded [2]. While fever, fatigue, and dry cough are commonly experienced upon SARS-CoV-2 infection [3], severe COVID-19 symptoms such as pneumonia, acute respiratory distress syndrome (ARDS), and multiple organ failure occur in approximately 15% of the patients and are mainly caused by a virus infection-induced cytokine storm [4, 5]. Multiple vaccines have been licensed less than 18 months after the first reported case [6–9], making the global vaccination campaign against COVID-19 an unprecedented success. Nevertheless, a problem occurring during the ongoing pandemic is the emergence of mutated SARS-CoV-2 variants (e.g., α-B1.1.7, β-B1.351, δ-B1.617.2), which exhibit a reduced susceptibility to vaccination [10, 11]. Moreover, antivirals are still sparse, with only a few having received emergency or compassionate use licensing, despite tremendous efforts [9, 12–14].

Because disease progression is dynamic, with late stages of severe COVID-19 driven by a harmful hyperinflammatory response, direct targeting antivirals (DTA) such as Remdesivir or monoclonal antibodies cannot efficiently improve health of severely diseased patients, albeit showing good effects when administered early in infection [13, 14].

Moreover, DTA pose the risk of emerging resistant virus strains, as with the M2 ion channel inhibitor Amantadine that rapidly forced influenza virus to develop a fully resistant phenotype [15]. In this regard, host targeting antivirals (HTA) directed against cellular factors that are required for viral life cycle might be advantageous, due to the inability of the virus to circumvent the affected cellular function. In the last decade, replication of DNA [16–18] and RNA viruses [15, 19–25] has been found to rely on the Raf/MEK/ERK signaling pathway which is involved in a vast variety of cellular processes including cell proliferation, differentiation, and survival [26], suggesting that this signaling axis is a promising antiviral target. Importantly, SARS-CoV spike (S) protein affects calcium-dependent activation of PKCα and promotes COX-2 protein synthesis via Raf/MEK/ERK activation [27]. Moreover, genomic and subgenomic RNA synthesis of the murine betacoronavirus mouse hepatitis virus (MHV) can be blocked through inhibition of MEK1/2 [28]. Encouraged by these findings, we thus explored the antiviral capacity of ATR-002 (Zapnometinib), an inhibitor of the two kinase isoforms MEK1 and MEK2, in the SARS-CoV-2 infection scenario. ATR-002 and its parental drug CI-1040 inhibit the replication of influenza viruses in vitro and in vivo [21, 29]. A recent phase 1 clinical trial in healthy individuals has already confirmed that ATR-002 is safe and well tolerated (ClinicalTrials.gov Identifier: NCT04385420), paving the way for the planned phase 2 trial to treat influenza in hospitalized patients. In addition to the direct effect on viral replication, both ATR-002 and CI-1040 are immunomodulatory, decrease the expression of pro-inflammatory cytokines/chemokines [20, 30], and thus might prevent the life-threatening hyperinflammation observed in patients suffering from severe COVID-19. In this study, we report the impact of the Raf/MEK/ERK signaling on SARS-CoV-2 replication and evaluate the anti-SARS-CoV-2 activity of the MEK1/2-inhibitor ATR-002 to counterbalance virus-induced pro-inflammatory cytokine/chemokine expression.

## Materials and methods

### Cell lines and human primary nasal airway epithelial cells (AECs)

Human airway epithelial cells (Calu3) from ATCC were taken from the cell line collection of the IVM. The cells were grown in Dulbecco’s modified Eagle medium/Nutrient Mixture F12-Ham (DMEM/F12-HAM) (Sigma-Life Science) supplemented with 10% (v/v) fetal bovine serum (FBS) (Capricorn Scientific) and 1% (v/v) Penicillin/Streptomycin (P/S) (Sigma-Life Science).

Human alveolar lung epithelial cells (A549) from ATCC, African green monkey kidney epithelial cells (VeroE6) and human embryonic kidney cells (HEK293T) from the cell line collection of the IVM were grown in Dulbecco’s modified Eagle medium (DMEM) (Sigma-Life Science) supplemented with 10% (v/v) FBS.

TMPRSS2-expressing A549 cells were obtained from the German Primate Center in Goettingen. The cells were grown in DMEM supplemented with 10% (v/v) FBS, 1% (v/v) P/S, and 1 µg/ml puromycin (InvivoGen).

TMPRSS2-expressing Vero76 cells were obtained from the German Primate Center in Goettingen. The cells were grown in DMEM supplemented with 10% (v/v) FBS, 1% (v/v) P/S, and 10 µg/ml blasticidin (Roth).

Human ACE2-expressing A549 cells were obtained from the German Primate Center in Goettingen. The cells were grown in DMEM supplemented with 10% (v/v) FBS, 1% (v/v) P/S, 1 µg/ml puromycin, 1% (v/v) non-essential amino acid solution (NEAA) (Sigma-Life Science), and 1% (v/v) sodium pyruvate solution (Gibco).

Human ACE2- and TMPRSS2-expressing A549 cells were obtained from the German Primate Center in Goettingen. The cells were grown in DMEM supplemented with 10% (v/v) FBS, 1% (v/v) P/S, 1 µg/ml puromycin, 1 µg/ml blasticidin, 1% (v/v) NEAA solution, and 1% (v/v) sodium pyruvate solution.

Human colorectal adenocarcinoma cells (CaCo2) were obtained from the Department of Immunology in Tuebingen. The cells were grown in DMEM supplemented with 10% (v/v) FBS, 1% (v/v) P/S, and 1% (v/v) non-essential amino acid solution (NEAA) (Sigma-Life Science).

Human primary nasal AECs (Table S2) were harvested from healthy adult volunteer donors by nasal brushing of the inferior turbinate and processed for cultivation at the air–liquid interface (ALI) as described previously [31]. Briefly, immediately after collection, the sampling brush was agitated to detach cells from the brush tip into 5 ml of serum-free isolation medium (Minimum Essential Medium, MEM) (Thermo Fisher Scientific) supplemented with 100 U/ml P/S (Lonza), 0.25 µg/ml amphotericin B (Sigma-Life Science), 50 µg/ml gentamicin (Sigma-Life Science), and 100 U/ml nystatin (Thermo Fisher Scientific). Subsequently, AECs were seeded at a concentration of 5 × 10^4^/ml in epithelial cell serum-free growth medium (BEGM; LHC basal medium) (Thermo Fisher Scientific) plus additives [31] supplemented with 1% P/S and 50 µg/ml gentamicin (from day 6 on without gentamicin) into culture flasks precoated with collagen solution from human fibroblasts (Sigma-Aldrich). Media were changed 24 h after seeding and thereafter every 3 days. After 7–10 days of culture, after reaching about 70–90% confluence, cells were detached with trypsin–EDTA 0.05% (Gibco) for 5 min and seeded apically on Transwell permeable supports (6.5 mm diameter; 0.4 μm pore size) (Corning) in ALI medium (LHC basal medium and DMEM at 1:1 plus additives [31] supplemented with 1% P/S) at a density of 50,000 cells in 200 µl ALI medium per Transwell. About 500–600 µl ALI medium was placed at the basolateral side and changed every other day. Once the cells had reached full confluency (after 5–7 days), they were induced to differentiate at ALI by removing medium at the apical side of the Transwell. After 21 days of culture at ALI, well-differentiated AECs were used for virus infection.

All cells were incubated at 37 °C and 5% CO_2_ atmosphere.

### Virus isolation

SARS-CoV-2 sputum samples were obtained from the Department of Clinical Virology of the University Hospital Muenster, after determination of the SARS-CoV-2 virus variant by sequencing. Sputum samples were centrifuged for 15 min at 4 °C and supernatants were diluted in cell culture medium, followed by filtration through a 0.5 µm syringe filter. Vero76-TMPRSS2 cells were incubated with filtered samples for 2 h at 37 °C. Afterward, cells were incubated in cell culture medium containing 2 × Antibiotic–Antimycotic (Gibco) for 96 h. Titers were determined by plaque titration. The passaging was repeated until viral concentrations were high enough to conduct experiments. Viral stocks were sequenced to exclude viral genome mutations caused by the cell culture propagation.

### Virus propagation

SARS-CoV-2 viruses were handled in a laboratory approved for biosafety level (BSL) 3 work. Viruses were propagated on Vero76-TMPRSS2 cells diluted in DMEM supplemented with 2% (v/v) FBS, 1% (v/v) P/S, 1% (v/v) sodium pyruvate solution, 1% (v/v) NEAA solution, and 1% (v/v) HEPES solution (Sigma-Aldrich) (Infection-DMEM) using an MOI of 0.01. 3 days post-infection (p.i.), the supernatant was collected and the virus titer was determined by plaque titration.

### Virus infection

SARS-CoV-2 viruses (Table S1) were diluted in cell culture medium according to the desired MOI. Cells were washed twice with PBS (Sigma-Aldrich) and incubated with the viral dilutions for 1 h at 37 °C. Cells were washed once with PBS and incubated in cell culture medium.

Primary AECs were washed twice with HBSS (Thermo Fisher Scientific) and incubated apically with the viral dilutions for 2 h at 37 °C. Cells were washed twice with HBSS and incubated at ALI.

After the indicated total incubation times, supernatants were collected and cells were subjected to the respective analysis method.

### Inhibitors and treatment

The MEK1/2-inhibitor ATR-002 (PD184264, Zapnometinib) (Atriva Therapeutics GmbH) was dissolved in DMSO (Roth) and used in final concentrations of 1–600 µM. DMSO served as control, using a final concentration of 0.1%.

Inhibitor treatment was performed as described in the respective experiments.

### siRNA knockdown

ERK1/2 was knocked down in Calu3 cells using Lipofectamine^®^ 2000 (Invitrogen) according to the manufacturer’s protocol.

48 h prior to infection, the cells were transfected with the siRNAs against ERK1/2 (siERK1/2) (Cell Signaling), or scrambled control siRNA (siCtrl) (Cell Signaling). 24 h post-transfection (p.t.) medium was changed.

### Cell cytotoxicity assay

Calu3 cells were grown in 96-well flat-bottom tissue plates. 24 h after seeding cells were incubated with different inhibitor concentrations (Table S3) for 24 h or 72 h. 15 µl sterile water or 10% Triton X-100 solution (CytoSelect™ LDH Cytotoxicity Assay Kit) (Cell Biolabs Inc.) was added and incubated for 10 min at room temperature. Collected supernatants were frozen at − 80 °C until measurement. 10 µl of LDH Cytotoxicity Assay Reagent (CytoSelect™ LDH Cytotoxicity Assay Kit) was mixed with 90 µl supernatant in a clear cell culture plate suitable for a plate reader. Analysis was performed using an ELISA reader (BioTek) at 405 nm after the samples were incubated at 37 °C and 5% CO_2_ for 20 min.

### Virus titration by plaque assay

Tenfold dilution series of SARS-CoV-2 containing solutions in PBS supplemented with 1% (v/v) P/S, 0.6% (v/v) BSA (35%) (Sigma-Aldrich), 0.01% (w/v) CaCl_2_ (Roth), and 0.01% (w/v) MgCl_2_ (Roth) was prepared to infect VeroE6 cells grown to a confluent monolayer in 6-well plates. 1 h.p.i. at 37 °C the virus inoculum was replaced by plaque medium composed of 63% (v/v) 2 × MEM ((20% (v/v) 10 × MEM (Gibco), 3.2% (v/v) NaHCO_3_ (7.5%) (Gibco), 2% (v/v) HEPES (1 M; pH 7.2) (Sigma-Aldrich), 1.2% (v/v) BSA (35%), 1% (v/v) 100 × Penicillin/Streptomycin/l-Glutamine solution (10,000 U/ml Penicillin; 10,000 µg/ml Streptomycin; 29.2 mg/ml l-Glutamine) (Gibco)), 2% (v/v) FBS, and 35% (v/v) Agar (2%) (Oxoid). Plaques were counted after 72 h incubation at 37 °C.

### Western blot analysis

Cellular and viral proteins were analyzed by Western blot. After a wash step with PBS, cells were lysed in RIPA (radioimmunoprecipitation) buffer (137 mM NaCl (Roth), 25 mM Tris–HCl (pH 8) (Roth), 2 mM EDTA (pH 8) (Roth), 10% (v/v) glycerol (Roth), 1% (v/v) NP-40 (Sigma-Aldrich), 0.5% (w/v) sodium deoxycholate (Serva), 0.1% (w/v) SDS (Roth)) supplemented with protease inhibitors (1:1000 Pefablock (200 mM) (Roth), 1:1000 Leupeptin (5 mg/ml) (Serva), 1:1000 Aprotinin (5 mg/ml) (Roth), 1:100 Na_3_VO_4_ (100 mM) (Sigma), and 1:200 Benzamidin (1 M) (Sigma)) centrifuged at 4 °C and 20,000 rpm for 10 min. Cleared lysates were mixed with 5 × Laemmli buffer and headed for 2 min at 95 °C. Protein separation was performed by SDS-PAGE and protein transfer onto nitrocellulose membranes by Western blotting, followed by blocking in TBS-T buffer (150 mM NaCl, 50 mM Tris–HCl (pH 7.5), 0.2% Triton X-100 (Roth), 1% Tween-20 (Roth)) containing 3% BSA (w/v) (Roth) for 1 h. Membranes were incubated for 1 h or overnight with primary antibodies (Table S1) diluted in blocking buffer. A 45 min incubation with 1:3000 secondary antibody (Table S1) dilutions in TBS-T was performed to detect the primary antibodies using chemiluminescence and the Li-CorOdissey^®^ Fc Imaging System. Signals were analyzed by the Image Studio™ (LiCor) software.

### Indirect immunofluorescence

Cells on glass coverslips were fixated at 4 °C for 10 min with ice-cold methanol (Roth) (− 20 °C), washed once with PBS, and blocked in 3% (w/v) BSA in PBS at room temperature for 1 h. Monoclonal anti-SARS-CoV-2 nucleocapsid (*N*) (SinoBiologicals, 1:1000) antibody was diluted in blocking buffer and cells were incubated at room temperature for 1 h, prior washing and 45 min incubation with secondary antibody Alexa568-conjugated (Invitrogen, 1:600) and DAPI (5 mg/ml) (Invitrogen, 1:10,000). Mounting of coverslips was performed using Fluorescence Mounting Medium (Dako). The Axiovert 200 M microscope and the AxioVision V4.8.2.0 (Zeiss) software were used to capture and analyze the epifluorescence pictures.

### Quantitative real-time PCR

Total RNA was isolated using the RNeasy Mini Kit (Qiagen) according to the manufacturer’s protocol. cDNA was synthesized mixing 1 µg RNA, Revert AID H Minus Reverse Transcriptase (Thermo Fisher Scientific), and oligo (dT) primers (Eurofins MWG Operon). The qRT-PCR reaction mix Brilliant III SYBR Green QPCR Master Mix (Agilent Technologies), specific primers (Table S1), and the LightCycler^®^ 480 II (Roche) in combination with the program LightCycler^®^ 480 SW V1.5.1.62 were used for the qRT-PCR analysis. GAPDH was used as reference.

### LEGENDplex™ multiplex assay

Cytokine and chemokine secretion was analyzed using the LEGENDplex™ Multiplex Assay (BioLegend^®^) according to the manufacturer’s protocol. Cells were transfected with 0.5 µg polyI:C (InvivoGen) using Lipofectamine^®^ 2000 (Invitrogen), followed by an incubation with different concentrations of ATR-002. 24 h.p.t. cytokine and chemokine amounts were measured using the LEGENDplex™ Human Anti-Virus Response Panel (13-plex) in combination with the Flow cytometer Gallios™ (Beckman Coulter).

### HTRF-based quantification of MAPK/ERK1/2 phosphorylation levels

To quantify the levels of pERK1/2 (Thr202/Tyr204) in mock or SARS-CoV-2-infected Calu3 cells, a plate-based assay (Cisbio) was used according to the manufacturer’s protocol. Briefly, cells grown on 96-well plates (50,000 cells/well) were non-infected or infected with different SARS-CoV-2 isolates for 1 h at 37 °C. Lysates were then transferred to a 384-well low-volume plate, incubated with the antibodies for 4 h at room temperature, and luminescence was recorded with a CLARIOstar reader (BMG Labtech) (200 flashes/well, integration start 60 µsec, integration time 400 µsec, and settling time 100 µsec). HTRF ratios were normalized to maximum system output obtained through stimulation with 100 nM phorbol 12-myristate 13-acetate (PMA) (AppliChem).

### VSV-pseudotyped system

SARS-CoV-2 S protein receptor-mediated entry was analyzed with the SARS-CoV-2 S protein-bearing vesicular stomatitis virus (VSV) pseudotyped system, containing a G gene substitution by a green fluorescence protein gene (VSV ΔG/GFP-Luc + S Δ21). The pseudotyped virus was generated according to Berger Rentsch and Zimmer, 2011 [32]. 24 h after transfection of the S coding plasmid into HEK293T cells, the inoculation with VSV ΔG + G-trans-complemented virus particles was initiated (provided by G. Zimmer). After 1 h, incubation cells were washed with PBS and treated with culture medium supplemented with anti-VSV-G antibody produced by I1-hybridoma cells to inactivate residual VSV-G particles. After 18 h incubation, the supernatant was collected and centrifuged through a 100 kDa spin column to concentrate the produced virus. Afterward, virus titration was performed in VeroE6 cells using the virus carrying GFP reporter to obtain FFU/ml.

Cells were infected with the pseudotyped VSV ΔG/GFP-Luc + S Δ21 using an MOI of 0.003 for 1 h. ATR-002 (100 µM) treatment was initiated simultaneously with the infection (0 h) or 1 h post-infection. The solvent DMSO served as control. 16 h.p.i. GFP-positive cells were quantified by light microscopy using the Axiovert 200 M microscope (Zeiss).

### Quantification and statistical analysis

Western blot signal intensities were measured using the LiCor Image Studio™ version 5.2.5 (LiCor). Immunofluorescence pictures were formatted using the AxioVersion V4.8.2.0 software (Zeiss) and quantified with ImageJ 1.48v (NIH Image). LEGENDplex data were analyzed using the LEGENDplex™ Data Analysis Software version 8.0.

GraphPad PRISM version 8.4.3 (GraphPad Software) was used to create the shown graphs and perform statistical analysis. Information about sample size and statistical tests are shown in the respective figure legends.

## Results

### SARS-CoV-2 infection triggers an early monophasic activation of the Raf/MEK/ERK signaling pathway

**Fig. 1:**
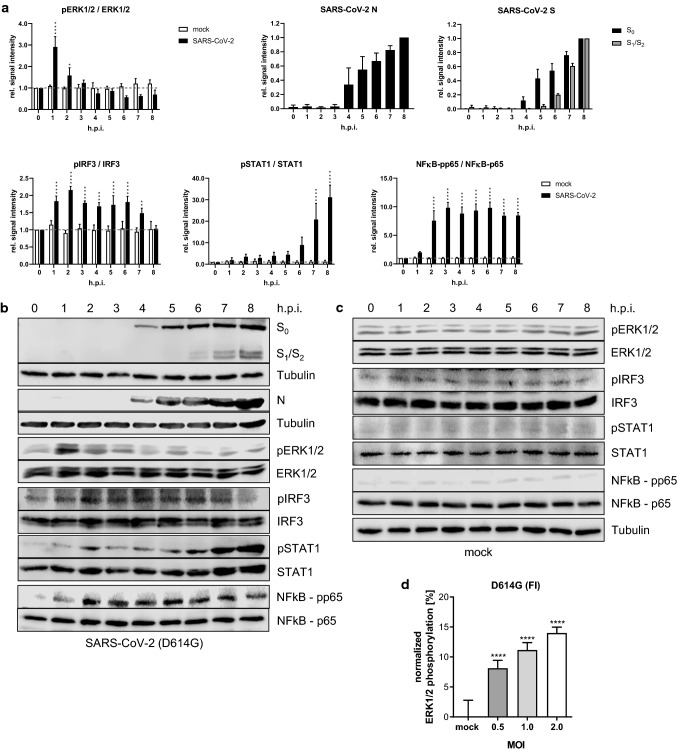
SARS-CoV-2 activates ERK1/2 in the early phase of the infection. **a–c** Calu3 cells were infected with SARS-CoV-2 (D614G-FI) (MOI 2). Mock-infected cells served as control. Western Blot lysates were prepared after the indicated time points. *See also Fig. S1c.* **a** Quantification of kinase phosphorylation and SARS-CoV-2 protein expression during the time course of infection. 0 h served as reference for pERK1/2, pIRF3, pSTAT1, and NFκB-pp65. 8 h served as reference for the viral proteins N and S. Reference time points were arbitrarily set to 1.0. Dashed lines indicate 0 h intensity. **b, c** Exemplary Western Blot analysis of (**a**). **d** Calu3 cells were infected with SARS-CoV-2 (D614G-FI) using indicated MOIs. ERK1/2 phosphorylation was analyzed 1 h.p.i. Mock served as reference. *See also Fig. S1a, b.* **a, d** Shown are means ± SD of three independent experiments. Data passed an one-way ANOVA followed by Dunnett’s multiple comparison test (*p ≤ 0.0332; **p ≤ 0.0021; ***p ≤ 0.0002; ****p ≤ 0.0001)

We first assessed whether SARS-CoV-2 infection activated the Raf/MEK/ERK kinase cascade. Western blot analysis of SARS-CoV-2 (D614G-FI)-infected Calu3 cells revealed a prominent ERK1/2 activation in the early phase of the infection, with a peak of ERK1/2 phosphorylation 1 h post-infection (h.p.i.), followed by a decrease over time (Fig. 1a–c). To explore the correlation between viral titers and ERK1/2 activation, we infected Calu3 cells with various multiplicities of infection (MOI: 0.5, 1.0, 2.0) and analyzed the changes in ERK1/2 phosphorylation in a homogeneous time-resolved fluorescence (HTRF)-based assay. A significant virus concentration-dependent increase in ERK1/2 phosphorylation was seen 1 h.p.i. (Fig. 1d; Fig. S1a, b). We included a Wuhan-like wild type isolate (WT), additional D614G isolates (LP, NK), and variants of concern (VOC) (α-B1.1.7, β-B1.351) to exclude a strain- or variant specific effect (Fig. S1a, b). While the activation of the Raf/MEK/ERK signaling cascade seemed to be crucial during the very early phase of infection, a second activation phase of the pathway could not be detected at later stages. At 4 h.p.i., the time point at which viral protein expression (S, N) was detected, the ERK1/2 phosphorylation intensity dropped below the level observed in mock-infected cells (Fig. 1a–c). The antiviral interferon (IFN) response was induced 1 h.p.i. represented by an increase in the phosphorylation of the interferon regulatory factor 3 (IRF3) and the inflammatory response was activated 2 h.p.i. indicated by an induction in the NFκB-p65 phosphorylation (Fig. 1a–c) Between 5 and 6 h.p.i., first virus particles were released and signal transducer and activator of transcription (STAT1) phosphorylation occurred, a prerequisite for the onset of the antiviral IFN response (Fig. 1a–c; Fig. S1c).

### The MEK1/2-inhibitor ATR-002 inhibits SARS-CoV-2 replication

**Fig. 2:**
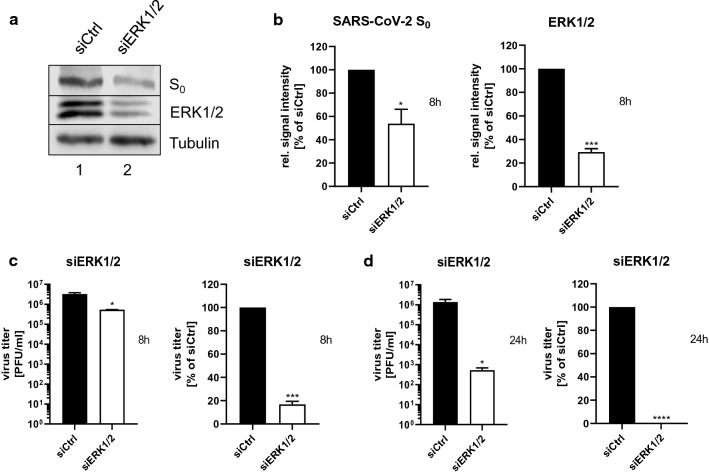
ERK1/2-knockdown results in decreased production of progeny viral titers. An ERK1/2-knockdown was introduced in Calu3 cells. 72 h.p.t. cells were infected with SARS-CoV-2 (D614G-FI) (**a**-**c**: MOI: 1; **d**: MOI: 0.1). **a** Expression of the viral S_0_ protein was analyzed 8 h.p.i. Immunoblots were prepared and probed with anti-S, anti-ERK1/2, and anti-Tubulin antibodies. Shown are results of one out of three independent experiments. **b** Quantification of the S_0_ protein expression and the knockdown efficacy. Shown are means ± SD of three independent experiments. **c** Titers of (**a**). **d** Titer analysis 24 h.p.i. **b, c, d** Percentage: siCtrl was arbitrarily set to 100%. Data passed a paired two-tailed *t* test (*p ≤ 0.0332; ***p ≤ 0.0002; ****p ≤ 0.0001). **c, d** PFU/ml: Data passed an unpaired two-tailed *t* test with Welch correction (*p ≤ 0.0332). PFU/ml and percentage: Shown are means ± SD of three independent experiments, each performed in duplicates

To confirm the importance of the ERK1/2-activation for the SARS-CoV-2 life cycle, we knocked down ERK1/2 in Calu3 cells via small interfering RNA (siRNA)-mediated RNA interference (RNAi), with a knockdown efficacy of 70.5% ± 2.9% (Fig. 2a, b). 72 h post-transfection (h.p.t.), the cells were infected and viral protein expression as well as viral titers were analyzed at 8 h.p.i, revealing a diminished S protein expression of 46.25% ± 12.4% and a concomitant reduction in the production of progeny viral particles of approximately one log unit (83.2% ± 2.6%) (Fig. 2b, c). The antiviral effect of the ERK1/2-knockdown was even higher in multi-cycle viral growth kinetics. 24 h.p.i. viral titers were approximately 3 log units (99.95% ± 0.02%) lower in knockdown cells compared to control cells (Fig. 2d). These results strongly support the relevance of ERK1/2 signaling in the SARS-CoV-2 life cycle. Because ERK1/2 is phosphorylated exclusively by ERK1/2 kinases MEK1/2 [33], we next explored the anti-SARS-CoV-2 properties of the MEK1/2-inhibitor ATR-002 against different SARS-CoV-2 variants. Calu3 cells were treated with increasing amounts of ATR-002 (1–200 µM) and the release of progeny viral particles was analyzed 24 h.p.i. by plaque titration. The ATR-002 concentration required for 50% inhibition (IC_50_) was in a range of 15.8–37.21 µM for the different SARS-CoV-2 variants including the VOCs α-B1.1.7, β-B1.351, and δ-B1.617.2 (Fig. S2a). The cytotoxic concentration that caused 50% cell death (CC_50_) was 895.0 µM (Fig. S2b). The resulting very favorable selectivity indices (SI) between 24.05 and 56.64 (Table 1) confirmed that ATR-002 activity was selective toward the viruses and not the host.

**Table 1:** IC_50_ and IC_90_ validation of the MEK1/2 inhibitor ATR-002 for different SARS-CoV-2 virus variants

Virus	log IC_50_ [µM]	IC_50_ [µM]	SI_IC50_	log IC_90_ [µM]	IC_90_ [µM]
Wild type (WT)					
München-1/02/2020/984	1.199	**15.80**	56.64	1.526	**33.60**
D614G					
FI hCoV-19/Germany/FI1103201/2020	1.496	**31.33**	28.57	1.870	**74.13**
NK hCoV-19/Germany/NK110320/2020	1.569	**37.08**	24.13	1.930	**85.04**
LP hCoV-19/Germany/LP110320/2020	1.571	**37.21**	24.05	1.898	**79.03**
Variants of concern					
α-B1.1.7—UK hCoV-19/Germany/NW-RKI-I-0026/2020	1.406	**25.47**	35.14	1.876	**75.17**
β-B1.351—South Africa hCoV-19/Germany/NW-RKI-I-0029/2020	1.481	**30.30**	29.54	1.833	**68.00**
B1.177.81—VOC from Spain hCoV-19/Germany/202105/2021	1.451	**28.22**	31.72	1.880	**75.81**
δ-B1.617.2—Indian hCoV-19/Germany/326763/2021	1.306	**20.25**	44.20	1.812	**64.90**

The IC50 values refer to the IC50 calculation in the supplementary data Fig. S2a. Within the supplementary data the R2-values for the IC50 values are given in bold

Notably, 72 h.p.i. viral titers (D614G-FI) were reduced by a half log unit at 50 µM ATR-002 up to 6.5 log units at 150 µM ATR-002, demonstrating a sustained inhibitory impact on the viral life cycle at non-cytotoxic concentrations (Fig. S2c, d). A cytopathic effect (CPE) caused by viral infection was first detected 48 h.p.i. which was not visible upon treatment with 50 µM ATR-002. Higher ATR-002 concentrations of 100 µM and 150 µM prevented CPE even 72 h.p.i. (Fig. S2e). Together, these results strongly support the suitability of the MEK1/2-inhibitor ATR-002 as a host targeting antiviral.

### The Raf/MEK/ERK signaling pathway plays a role in the early phase of the SARS-CoV-2 life cycle

**Fig. 3:**
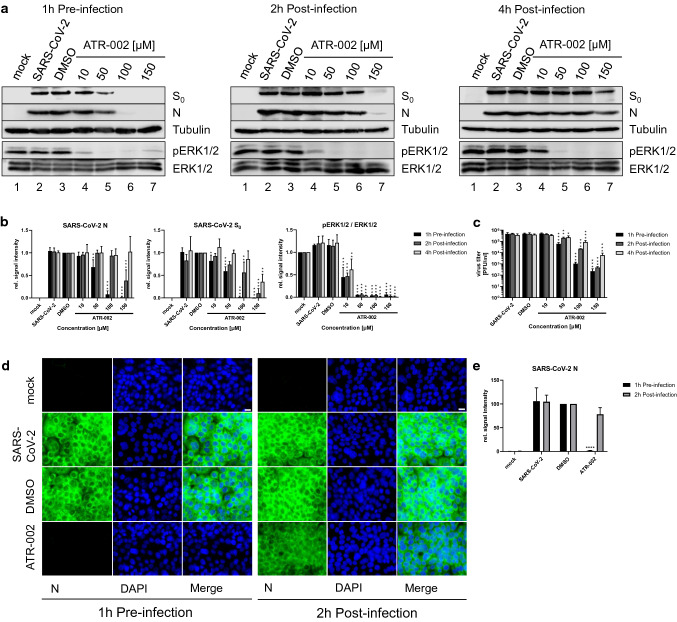
Time-dependent inhibitory effect of ATR-002 on the SARS-CoV-2 replication cycle. Calu3 cells were infected with SARS-CoV-2 (D614G-FI) (MOI 2). **a** ATR-002 (10–150 µM) treatment was initiated 1 h pre-infection, 2 h post-infection, or 4 h post-infection. Immunoblots were prepared 8 h.p.i. and probed with anti-S, anti-N, anti-pERK1/2, anti-ERK1/2, and anti-Tubulin antibodies. *See also Fig. S3a*. **b** Quantification of (**a**). Intensity ratios of S_0_ and N were normalized to Tubulin. Intensity ratios of pERK1/2 were normalized to the total protein amount. DMSO (S_0_, N) or mock (pERK1/2 / ERK1/2) was arbitrarily set to 1.0. *See also Fig. S3b*. **c** Viral titers of (**a**). *See also Fig. S3c*. **d** ATR-002 (100 µM) treatment was initiated 1 h pre-infection or 2 h post-infection. 8 h.p.i. samples were prepared for immunofluorescence analysis. Same laser and detector settings were used. Scale bar indicates 20 µm. **e** Intensity ratios of the SARS-CoV-2 N protein signals. DMSO was arbitrarily set to 100. **a, d** Mock infection, SARS-CoV-2 infection, and DMSO (0.1%) treatment served as controls. Shown are results of one out of three independent experiments. **b, c, e** Shown are means ± SD of three independent experiments. Data passed an one-way ANOVA followed by Dunnett’s multiple comparison test (*p ≤ 0.0332; **p ≤ 0.0021; ***p ≤ 0.0002; ****p ≤ 0.0001) for each time point separately

We next addressed the question whether the antiviral activity of ATR-002 was limited to the early phase of the SARS-CoV-2 infection cycle. Therefore, cells were treated with ATR-002 either 1 h pre-infection or 2 h/4 h post-infection and the expression of viral proteins (S, N) and progeny viral titers were determined 8 h.p.i. (Fig. 3a–c; Fig. S3). While the ERK1/2 phosphorylation was reduced by approximately 50% for the 10 µM ATR-002 concentration, higher amounts (50–150 µM) resulted in an almost complete inhibition of the phosphorylation signal. Interestingly, the expression of N protein remained unchanged, and S protein expression was only marginally reduced (18% ± 0.13%) when cells were treated with 10 µM of ATR-002 1 h pre-infection (Fig. 3b; Fig. S3b). In addition, no changes in viral titers were found, indicating that the remaining ERK1/2 activity was sufficient to maintain its role in the viral life cycle (Fig. 3c; Fig. S3c). A 10 µM ATR-002 treatment 2 h.p.i. and 4 h.p.i. did also not affect the protein expression and viral particle release. A significant reduction of viral protein expression and viral titers could be found at ATR-002 concentrations ranging from 50 to 150 µM, when treatments were started 1 h pre-infection (Fig. 3a–c; Fig. S3). In contrast, the inhibitory effect was less pronounced in the post-infection scenarios. Viral protein expressions started to occur for 100 µM 2 h.p.i. and for 150 µM 4 h.p.i. (Fig. 3a, b; Fig. S3a, b). In addition, viral titers increased with delayed ATR-002 treatment (Fig. 3c; Fig. S3c). Immunofluorescence analysis of the SARS-CoV-2 N protein expression confirmed these results (Fig. 3d, e). Comparable to the data shown in Fig. 3a–c, a strong inhibitory effect on the N expression was found for the 1 h pre-infection treatment but not for the 2 h.p.i. treatment. These data indicate that a very early activation of the Raf/MEK/ERK signaling is an important requirement for optimal viral replication.

### ATR-002 restricts SARS-CoV-2 replication in different cell lines albeit with variable efficiency

**Fig. 4:**
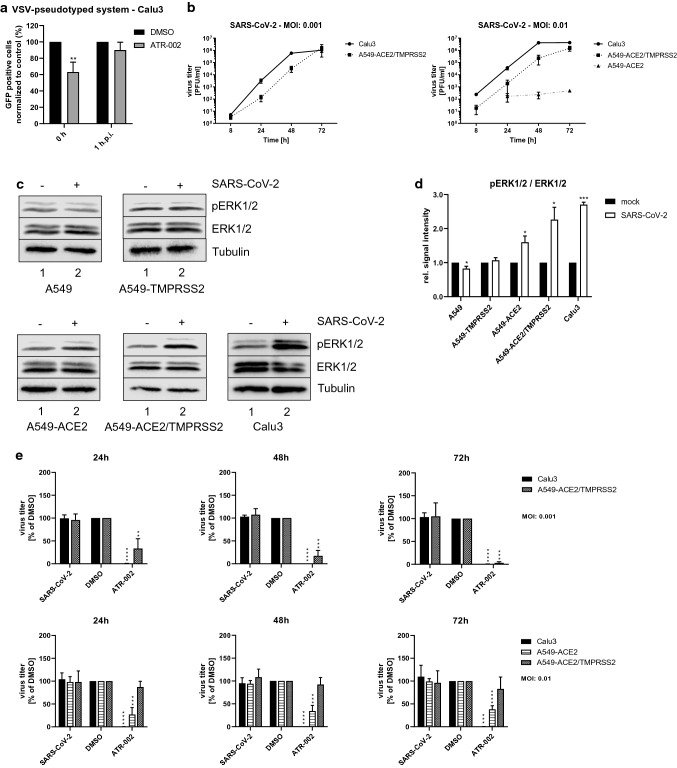
Inhibition of MEK1/2 by ATR-002 results in decreased viral titers in Calu3 and A549-ACE2 cells. **a** Calu3 cells were infected with the VSV-pseudotyped system VSV ΔG/GFP-Luc + S- Δ21. 0 h or 1 h.p.i. ATR-002 treatment was initiated. Infected cells were incubated for 16 h in the presence of ATR-002. **b, e** Depicted cell lines were infected with SARS-CoV-2 (D614G-FI) (MOI 0.001; 0.01). **b** Growth kinetic of SARS-CoV-2 (D614G-FI) in different cell lines. *See also Fig. S4b, e, h, k, n*. **c** Depicted cell lines were infected with SARS-CoV-2 (D614G-FI) (MOI 2) ( +). Immunoblots were prepared 1 h.p.i. and probed with anti-pERK1/2, anti-ERK1/2, and anti-Tubulin antibodies. Shown are results of one out of three independent experiments. Mock (-) infected cells served as negative control. **d** Quantification of (**c**). Intensity ratios of pERK1/2 were normalized to the total protein amount. Mock was arbitrarily set to 1.0. Data passed a paired two-tailed *t* test (*p ≤ 0.0332; ***p ≤ 0.0002) for each cell line separately. **e** Titer reduction of SARS-CoV-2 (D614G-FI) in different cell lines after MEK1/2-inhibition. 1 h.p.i. cells were treated with ATR-002 (100 µM). Untreated (SARS-CoV-2) and DMSO (0.1%) treated cells served as negative controls. Data passed an one-way ANOVA followed by Dunnett’s multiple comparison test (**p ≤ 0.0021; ***p ≤ 0.0002; ****p ≤ 0.0001) for each cell line separately. DMSO was used as reference and arbitrarily set to 100%. *See also Fig. S4c, d, f, g, i, j, l, m, o, p*. **a, b, d, e** Data represent means ± SD of three independent experiments, each performed in triplicates (**b**, **d**, **e**) or in quadruplicates (**a**)

To investigate a role of the early ERK1/2 activation during the viral internalization, we used the SARS-CoV-2 S protein-bearing vesicular stomatitis virus (VSV) pseudotype system, containing a G gene substitution by the green fluorescent protein gene (VSV Δ/GFP-Luc + S Δ21) [32, 34]. With this S-pseudotyped VSV, specifically designed to analyze the SARS-CoV-2 S protein receptor-mediated entry, we observed a significant reduction of GFP-positive Calu3 cells of 36.81% ± 6,9% if the inhibitor treatment was initiated together with viral infection (0 h), whereas a not significant reduction of 9.9% ± 5,5% was found if the inhibitor treatment was initiated 1 h.p.i. (Fig. 4a). Comparable results were found for the colorectal cancer cell line CaCo2, that serves as a model for an epithelial barrier (Fig. S4q). Both cell lines are described to endogenously express transmembrane serine protease 2 (TMPRSS2) [35] (Fig. S4a). A strongly reduced inhibitory effect on the SARS-CoV-2 S protein receptor-mediated entry was found in the Vero cell lines VeroE6 and Vero76-TMPRSS2, which do express high amounts of Cathepsin L (Fig. S4a, q). These results indicate that virus-induced ERK1/2 activation is important for the early TMPRSS2-mediated internalization process.

To assess whether SARS-CoV-2-mediated activation of Raf/MEK/ERK signaling was cell type specific, we next used A549 cells genetically modified to render them permissive to SARS-CoV-2 [35]. In the A549 cell line, expressions of both the main SARS-CoV-2 entry receptor Angiotensin-converting enzyme 2 (ACE2) and TMPRSS2 responsible for the priming of the spike protein [36] are only expressed at low levels (Fig. S4a). Hence, A549 cells are only marginally permissive for SARS-CoV-2 [37]*.* As expected, we could not detect progeny viral titers in the A549 cell lines lacking robust levels of endogenous ACE2 (parental A549 cells and A549-TMPRSS2 cells stably expressing TMPRSS2), whereas ACE2 overexpression allowed for a successful infection at MOI 0.01. At 48 h.p.i. and 72 h.p.i., viral titers were 3 log units and 4 log units, respectively, higher in ACE2/TMPRSS2-expressing A549 cells compared to A549-ACE2 cells. Highest viral titers were obtained in the naturally permissive Calu3 cells (Fig. 4b). For successful infection at lower MOIs of 0.001, the combined expression of ACE2 and TMPRSS2 was required (Fig. 4b). Notably, infection of A549-ACE2, A549-ACE2/TMPRSS2 and Calu3 cells using an MOI 2 led to a significant phosphorylation of ERK1/2 1 h.p.i. The activation intensity increased in A549-ACE2 cells by the factor 1.6 ± 0.18, in A549-ACE2/TMPRSS2 cells by the factor 2.26 ± 0.36, and in Calu3 cells by the factor 2.7 ± 0.07 compared to mock-infected cells. In line with their poor permissiveness, no increase in ERK1/2 phosphorylation was found for parental A549 and A549-TMPRSS2 cells (Fig. 4c, d). We additionally analyzed the ERK1/2 activation in VeroE6, Vero76-TMPRSS2, and CaCo2 cell lines. No ERK1/2 activation was found in the Cathepsin L expressing Vero cell lines, whereas in TMPRSS2-expressing CaCo2 cells, the ERK1/2 phosphorylation was increased by the factor 2.47 ± 1.46 (Fig. S4a, r, s).

The efficacy of ATR-002 to inhibit SARS-CoV-2 replication varied between the different cell lines, depending on the chosen MOI. Viral titers were significantly decreased in Calu3, A549-ACE2/TMPRSS2, and CaCo2 cells for all analyzed time points using an MOI of 0.001 (Fig. 4e; Fig. S4b–d, h–j, t). Comparable effects were found for Calu3, A549-ACE2, and CaCo2 cells using an MOI of 0.01 (Fig. 4e; Fig. S4e–g, n–p, u). No inhibitory effect was found for the Cathepsin L expressing Vero cell lines (Fig. S4t, u). Surprisingly, viral titers in A549-ACE2/TMPRSS2 cells were also not significantly decreased when the cells were infected with a MOI of 0.01 (Fig. 4e; Fig. S4k–m).

These results confirm that the antiviral activity of ATR-002 is not restricted to the Calu3 cell line and that activation of ERK1/2 is linked to efficient SARS-CoV-2 infection of cells via ACE2 and TMPRSS2. Nevertheless, the extent of antiviral activity seems to vary among cell lines with respect to the viral dose used for inoculation.

**Fig. 5:**
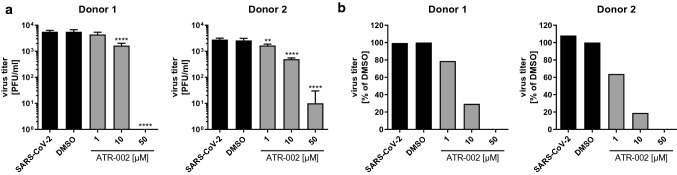
ATR-002 blocks production of progeny viral titers in human primary airway epithelial cells (AECs). Human primary nasal AECs were apically infected with SARS-CoV-2 (D614G-FI) (MOI 1). 2 h.p.i. cells were treated at the basolateral side with ATR-002 (1 µM, 10 µM, 50 µM) for 48 h followed by an incubation at the apical side for 20 min with 200 µl culture medium and supernatants were analyzed by plaque titration. Untreated (SARS-CoV-2) and DMSO (0.1%) treated cells served as negative controls. Data shows results of one experiment. *See also Fig. S5*. **a** Virus titer in PFU/ml. Data passed a one-way ANOVA followed by Dunnett’s multiple comparison test (**p ≤ 0.0021; ****p ≤ 0.0001). **b** Virus titer in percentage. DMSO was arbitrarily set to 100%

It is already known that lower amounts of ATR-002 are sufficient to achieve high inhibitory effects on the viral replication of Influenza A viruses in primary cells due to their lower basal ERK1/2 activity compared to immortalized cell lines [21]. To confirm these findings and investigate the role of ERK1/2 activation on SARS-CoV-2 infection in a more physiological scenario we next assessed the antiviral activity of ATR-002 in air–liquid-interface (ALI) cultures of primary nasal airway epithelial cells (AEC) which represent one of the most accurate models for infection of the upper respiratory tract. In this infection model, treatment with 1 µM ATR-002 already reduced viral titers by 20–40%. A similar reduction was only achieved in Calu3 cells upon treatment with 50 µM ATR-002, a concentration that fully inhibited virus production in the AEC cultures (Fig. 5; Fig. S5; Fig. S2a).

### ATR-002 dampens the induction of pro-inflammatory cytokine expression

**Fig. 6:**
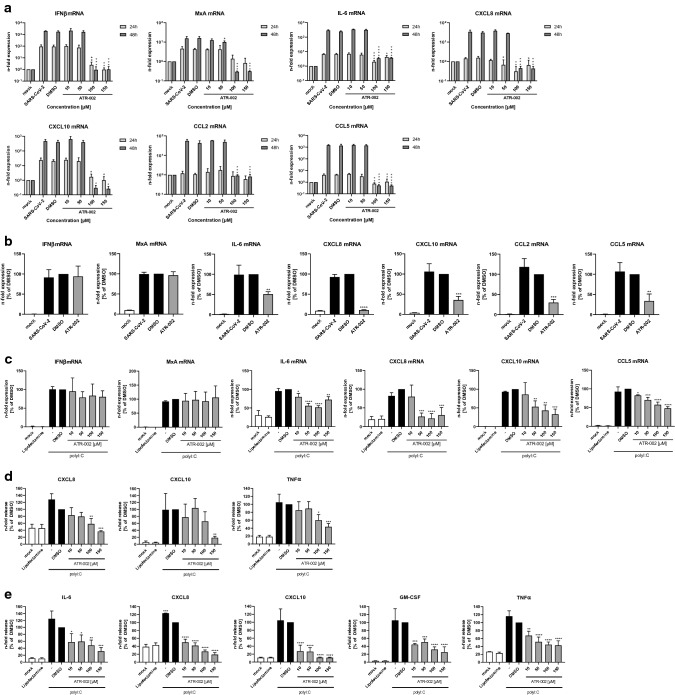
Inhibition of MEK1/2 by ATR-002 results in a decreased expression of pro-inflammatory cytokines/chemokines. **a, b** Calu3 cells (**a**) or A549-ACE2/TMPRSS2 cells (**b**) were infected with SARS-CoV-2 (D614G-FI) (MOI 0.01). 1 h.p.i. cells were treated with ATR-002 (**a**: depicted concentrations, **b**: 100 µM). mRNA expression was analyzed 24 h (**a**) and 48 h (**a**, **b**) p.i. Mock, SARS-CoV-2, and DMSO (0.1%) served as controls. *See also Fig. S6a*. **c–e** Calu3 cells (c, d) or A549-ACE2/TMPRSS2 cells (**e**) were transfected with polyI:C (100 ng/ml) and treated with ATR-002 for 24 h. Untreated (mock) and Lipofectamine treated cells served as negative controls, polyI:C stimulation and DMSO (0.1%) served as positive controls. **a–c** mRNA expression was determined by quantitative real-time PCR. Results are depicted as n-fold mRNA expression of mock-infected cells (**a**) or as n-fold expression of DMSO treated cells (**b**, **c**). Data represent means ± SD of three independent experiments, each performed in triplicates. **d, e** Cytokine and chemokine secretion was determined by flow cytometry. Results are depicted as *n*-fold release of DMSO treated cells. Data represent means ± SD of three independent experiments. *See also Fig. S6c, d.* **a–e** Data passed an one-way ANOVA followed by Dunnett’s multiple comparison test (*p ≤ 0.0332; **p ≤ 0.0021; ***p ≤ 0.0002; ****p ≤ 0.0001). **a** One-way ANOVA was conducted for each time point separately. **b–e** DMSO was used as reference and arbitrarily set to 100%

A hallmark of severe progression of COVID-19 is excessive lung damage that is mainly caused by acute inflammation and hypoxemia [38]. The resulting acute respiratory distress syndrome (ARDS) and extrapulmonary multi-organ dysfunction are provoked by the excessive production of pro-inflammatory cytokines (IL-6, CXCL8) and chemokines (CXCL10, CCL5, CCL2), a phenomenon also referred as cytokine storm [39]. Such exaggerated dysregulated cytokine response strongly increases the mortality rate in COVID-19 patients. An early and controlled alleviation of the cytokine storm can increase the likelihood of a mild course of the disease [39]. It could already be shown that MEK1/2-inhibitors have an inhibitory effect on the expression of pro-inflammatory cytokines/chemokines without interfering with the interferon-induced antiviral response [20, 30]. Therefore, the question arose if ATR-002 has a similar beneficial immunomodulatory effect during the course of a SARS-CoV-2 infection. Cytokine and chemokine expression analysis of the infection kinetic shown in Fig. S2d revealed a strong reduction of pro-inflammatory cytokines/chemokines, such as IL-6, CXCL8, CXCL10, CCL2, and CCL5, for ATR-002 concentrations of 100 µM and 150 µM (Fig. 6a; Fig. S6a). Because mRNA levels of the antiviral acting proteins IFNβ and MxA were also decreased in this assay, we hypothesized that a potential direct effect of ATR-002 on cytokine/chemokine expression was dominated here by the virus-inhibiting effect of ATR-002, leading to reduced stimulus. Thus, in a next step, we used the A549-ACE2/TMPRSS2 cells, which showed no inhibitory effect on viral titers after ATR-002 treatment when infected with a MOI of 0.01 (Fig. 4e; Fig. S4k–m), to exclude any effects caused by reduced viral growth. The IFN-mediated antiviral response was not altered in these cells. Comparable levels of IFNβ and MxA mRNAs were found for the controls (SARS-CoV-2, DMSO) and the ATR-002 treatment (100 µM). Despite the unchanged antiviral IFN response, pro-inflammatory cytokine and chemokine expression was significantly decreased. Strongest effects were found for CXCL8, with a reduction of 89.3% ± 0.86% compared to the DMSO control and weakest effects for IL-6 with a reduction of 49.5% ± 6.2% in mRNA expression (Fig. 6b). Comparable to the results in Fig. 4e, ATR-002 did not significantly reduce progeny viral titers (Fig. S6b). We furthermore aimed to analyze the direct inhibitory effect of ATR-002 on the expression of pro-inflammatory cytokines/chemokines in a virus-free system by transfecting and stimulating Calu3 cells with a synthetic analog of double-stranded RNA (polyI:C) mimicking viral RNA. This allows us to discriminate whether ATR-002 directly interferes with gene induction by a pathogen-associated molecular pattern (PAMP), because indirect effects due to reduced virus replication caused by ATR-002 in an infection scenario are avoided. No change in the cellular IFN response to the polyI:C stimulation was observed for the ATR-002 treatment using increasing amounts of the inhibitor (10–150 µM) (Fig. 6c). In contrast and in line with the results obtained in the A549-ACE2/TMPRSS2 cell line (Fig. 6b), expression of pro-inflammatory cytokines/chemokines was significantly decreased. The strongest effect was found for the expression of CXCL8 with a reduction of 78.2% ± 12.9% in cells treated with 100 µM of ATR-002; the weakest effects were observed for the expression of CCL5 and IL-6 upon treatment with 100 µM of ATR-002 with reductions of 42.5% ± 7.1% and 48.4% ± 4.4%, respectively (Fig. 6c). In an additional attempt, we aimed to analyze the effect of the ATR-002 treatment on the secretion of pro-inflammatory cytokines/chemokines. Therefore, we stimulated Calu3 or A549-ACE2/TMPRSS2 cells with polyI:C in a combinational treatment with ATR-002 (10–150 µM). Comparable to the mRNA expression levels in Fig. 6b, c, the MEK1/2 inhibition via ATR-002 reduced the secretion of the pro-inflammatory cytokines/chemokines IL-6, CXCL8, CXCL10, GM-CSF, and TNFα in a concentration-dependent manner (Fig. 6d, e; Fig. S6c, d).

These results strongly implied that the MEK1/2-inhibitor ATR-002 reduces the risk of a COVID-19-associated cytokine storm by attenuating the production of pro-inflammatory cytokine/chemokine expression.

## Discussion

A multitude of antiviral interventions against SARS-CoV-2/COVID-19 are under evaluation. However, the development of novel therapeutics including interferons [40], convalescent plasma treatment [41], new small-molecule drugs [42], and monoclonal antibodies [14] might require years. An alternative approach is the repurposing of already licensed drugs to target either viral components or cellular mechanisms involved in SARS-CoV-2 replication. In this study, we explored the involvement of the cellular Raf/MEK/ERK pathway in the SARS-CoV-2 infection, and assessed its antiviral potential and the suitability of the MEK1/2-inhibitor ATR-002 as a potential drug repurposing candidate. Our observation of a monophasic activation of ERK1/2 in the early phase of SARS-CoV-2 infection (Fig. 1; Fig. S1) adds to previous studies that already linked Raf/MEK/ERK signaling to the infection process of various viruses [15–23, 43, 44], including coronaviruses [27, 28, 45–47]. The significantly reduced virus production of all tested SARS-CoV-2 variants in cells in which this signaling pathway was blocked by either ERK1/2-knockdown or MEK1/2-inhibition (Figs. 2, 3, 4, 5; Table 1; Fig. S2–S5) confirms the potential as a druggable target, with efficient inhibitory effects of ATR-002, reflected by the low SI values. It is worth to mention that these values, which do reflect the ratio between the drug compatibility (CC_50_) and the inhibitory effect (IC_50_), are steady within the different virus isolates, indicating that the mutational changes of the SARS-CoV-2 variants did not influence the inhibitory ability of ATR-002. Inhibition of MEK1/2 signaling prior to SARS-CoV-2 infection was most effective, strongly arguing for an important role of the Raf/MEK/ERK pathway in the very early stage of the viral life cycle (Fig. 3; Fig. S3). This assumption is affirmed by the finding that entry of SARS-CoV-2 S-pseudotyped VSV was significantly reduced in case of ATR-002 treatment simultaneous with infection, whereas a 1 h lagged treatment did not show a significant entry reduction (Fig. 4a; Fig. S4q). As coronaviruses enter host cells either through a serine protease (e.g., TMPRSS2) dependent cell surface entry pathway or through an endocytic pathway using lysosomal cysteine proteases (Cathepsins), depending on the availability of serine proteases [48], the missing inhibitory effect of ATR-002 in A549-ACE2/TMPRSS2 cells when infected with higher viral titers (Fig. 4; Fig. S4) might suggest that Raf/MEK/ERK activation is connected to SARS-CoV-2 endocytosis, resembling the IAV-induced early ERK1/2 activation that stimulates V-ATPase-dependent endosomal acidification required for HA-mediated fusion of the viral envelope with the endosomal membrane [49]. However, we found only moderate inhibitory effects of ATR-002 on the SARS-CoV-2 S-pseudotyped VSV in Cathepsin L expressing VeroE6 and Vero76-TMPRSS2 cells, compared to cell lines for which we could not detect high amounts of Cathepsin L (Calu3, Caco2) (Fig. 4a; Fig. S4a, q). These findings are supported by the absence of the ERK1/2 activation in the Vero cell lines (Fig. S4r, s).

Another result pointing toward the involvement of TMPRSS2 is the high inhibitory ability of ATR-002 in primary human airway epithelial cells (AEC) (Fig. 5; Fig. S5), as it is already described that SARS-CoV-2 S-driven entry in AECs can efficiently be blocked with the TMPRSS2-inhibitor camostat mesylate [36]. If the Raf/MEK/ERK pathway would be related to the cathepsin pathway, strong inhibitory effects could be expected in the Cathepsin L expressing Vero cell lines, which we did not find in our experiments. Despite the evidence of the involvement of active ERK1/2 in the TMPRSS2-mediated cell surface entry pathway, we could not decipher the exact contribution of ERK1/2 in this process. Our data suggest that it might act as a cofactor or a posttranslational modifier for the entry process. Different studies have identified several cofactors that seem to play a role in the SARS-CoV-2 entry process. Besides TMPRSS2 and Cathepsin L [36], a contributing role of NRP-1 [50], all known to be modified by phosphorylation, as well as HSPGs [51] are described. Of note, except Cathepsin L, these cofactors are localized at the cell surface, where they directly interact with the spike protein. As intracellular kinase, a direct interaction of ERK1/2 during the binding process can be excluded. Nevertheless, active ERK1/2 or a downstream kinase may phosphorylate cofactors directed to plasma membrane, allowing for an indirect contribution to the internalization process.

Since hyperinflammation and multiple organ failure (lung, heart, kidney, liver, and brain) [5, 38, 39, 52] drive severe COVID-19 in late stages, pharmacological suppression of the pro-inflammatory cytokine/chemokine induction rather than interference with viral replication might be a promising treatment option. Most cytokines/chemokines are involved in immune cell activation and recruitment to the side of infection and inflammation; however, hyperactivation can have fatal consequences for the patients. MEK1/2-inhibition can reduce but not fully block the production of pro-inflammatory cytokines/chemokines (Fig. 6; Fig. S6) [20]. However, a drug should not completely suppress all cytokines/chemokines, since, e.g., induction of the antiviral interferon response is vital to mount an antiviral defense. As shown here and elsewhere, MEK1/2-inhibitors did not affect IFNβ—and interferon stimulated gene (ISG) mRNA expression such as, MxA. The data using a synthetic analog of vRNA (polyI:C) (Fig. 6c) and comparable results in A549-ACE2/TMPRSS2 cells (Fig. 6b) show that this is a direct effect on the induction mechanism of pro-inflammatory cytokines/chemokines and not indirectly caused via viral titer reduction.

In conclusion, the low efficiency of DTAs against acute, self-limiting and hyperinflammatory viral diseases, such as severe influenza and COVID-19, observed so far might call for a rethinking of antiviral concepts in general. A DTA attempt might be simply too late to rescue severely diseased patients in late stages of the disease. Here, host targeting antivirals (HTA) that at the same time have immunomodulatory effects, such as ATR-002, might offer a suitable toolbox for antiviral therapies of different viral origin (different viruses misuse similar cellular factors) without posing the risk of emerging resistances. Besides promising data on bioavailability [21] and safety of the compound (ClinicalTrials.gov Identifier: NCT04385420), a phase 2 clinical trial was initiated to verify the antiviral properties of ATR-002 (Zapnometinib) (RESPIRE: EudraCT:2020-004206-59) in intermediate stages of hospitalized COVID-19.

## Supplementary Information

Below is the link to the electronic supplementary material.

Supplementary file1 (DOCX 2872 KB)

## Data Availability

All data generated or analyzed during this study are included in this published article (and its supplementary information files).

## Abbreviations

ACE2: Angiotensin-converting enzyme 2
AEC: Airway epithelial cells
ALI: Air–liquid-interphase epithelial cell
ARDS: Acute respiratory distress syndrome
BEGM: Bronchial epithelial cell growth medium
BSA: Bovine serum albumin
BSL: Bio-safety level
CCL: Chemokine (C–C motif) ligand
COVID-19: Coronavirus disease 2019
COX-2: Cyclooxygenase-2
CPE: Cytopathic effect
CXCL: Chemokine (C–X–C motif) ligand
DMEM: Dulbecco’s modified Eagle medium
DMSO: Dimethyl sulfoxide
DTA: Direct targeting antivirals
EDTA: Ethylenediaminetetraacetic acid
ELISA: Enzyme-linked immunosorbent assay
ERK: Extracellular signal-regulated kinase
FBS: Fetal bovine serum
FFU: Focus-forming units
HA: Hemagglutinin
HBSS: Hank’s Balanced Salt Solution
HEPES: 4-(2-Hydroxyethyl)-1-piperazineethanesulfonic acid
HSPG: Heparan sulfate proteoglycan
HTA: Host targeting antivirals
HTRF: Homogeneous time-resolved fluorescence
IAV: Influenza A virus
IC50: 50% Inhibitory concentration
IFN: Interferon
IL: Interleukin
IRF3: Interferon regulatory factor 3
ISG: Interferon-stimulated gene
LDH: Lactate dehydrogenase
MCP-1: Monocyte chemoattractant protein-1
MEM: Eagle’s minimal essential medium
MEK: Mitogen-activated protein kinase
MHV: Murine betacoronavirus mouse hepatitis virus
MOI: Multiplicity of infection
mRNA: Messenger RNA
MxA: Myxovirus resistance protein-1
NEAA: Non-essential amino acids
NFκB: Nuclear factor kappa-light-chain-enhancer of activated B cells
NRP-1: Neuropilin-1
PBS: Phosphate-buffered saline
PFU: Plaque-forming units
P/S: Penicillin/Streptomycin
PKCα: Protein kinase C alpha
PMA: Phorbol 12-myristate 13-acetate
qRT-PCR: Quantitative real-time polymerase chain reaction
Raf: Rapid accelerated fibrosarcoma
RIPA: Radioimmunoprecipitation assay
RNAi: RNA interference
SARS-CoV-2: Severe acute respiratory syndrome coronavirus 2
SDS-PAGE: Sodium dodecyl sulfate polyacrylamide gel electrophorese
SI: Selectivity index
siRNA: Small-interfering RNA
STAT: Signal transducer and activator of transcription
TMPRSS2: Transmembrane serine protease 2
v-ATPase: Vacuolar-type ATPase
VOC: Variant of concern
vRNA: Viral RNA
VSV: Vesicular stomatitis virus
