# Supplementary material for: The MEK1/2-inhibitor ATR-002 efficiently blocks SARS-CoV-2 propagation and alleviates pro-inflammatory cytokine/chemokine responses
Source: Cell Mol Life Sci. 2022 Jan 10;79(1):65. doi: 10.1007/s00018-021-04085-1 (PMC8747446; doi:10.1007/s00018-021-04085-1)
Supplement: Supplementary file 1 — Supplementary file1 (DOCX 2872 KB) [file 18_2021_4085_MOESM1_ESM.docx]

**The MEK1/2-inhibitor ATR-002 efficiently blocks SARS-CoV-2 propagation and alleviates pro-inflammatory cytokine/chemokine responses**

**Journal:** Cellular and molecular life sciences

**Authors:** André Schreiber^a^, Dorothee Viemann^c-e^, Jennifer Schöning^c^, Sebastian Schloer^f^, Angeles Mecate Zambrano^a^, Linda Brunotte^a^, Aileen Faist^a,b^, Michael Schöfbänker^a^, Eike Hrincius^a^, , Helen Hoffmann^g,h^, Markus Hoffmann^i^, Stefan Pöhlmann^i^, Ursula Rescher^f,j^, Oliver Planz^g,h^, Stephan Ludwig^a,j,k,^*

**Affiliations:** ^a^ Institute of Virology (IVM), Centre for Molecular Biology of Inflammation, University of Muenster, Muenster, North Rhine-Westphalia, 48149, Germany

^b^ CiM-IMPRS Graduate School, University of Muenster, Muenster, North Rhine-Westphalia, 48149, Germany

^c^ Translational Pediatrics, Department of Pediatrics, University Hospital Wuerzburg, Bavaria, 97080, Germany

^d^ Center for Infection Research, University Wuerzburg, Bavaria, 97080, Germany

^e^ Cluster of Excellence RESIST (EXC 2155), Hannover Medical School, Lower Saxony, 30625, Germany

^f^ Research Group Regulatory Mechanisms of Inflammation, Institute of Medical Biochemistry, Centre for Molecular Biology of Inflammation, University of Muenster, Muenster, North Rhine-Westphalia, 48149, Germany

^g^ Atriva Therapeutics GmbH, Tuebingen, Baden-Württemberg, 72072, Germany

^h^ Interfaculty Institute for Cell Biology, Department of Immunology, Eberhard Karls University, Tuebingen, Baden-Württemberg, 72074, Germany

^i^ Infection Biology Unit, German Primate Center - Leibniz Institute for Primate Research, Goettingen, Germany; Faculty of Biology and Psychology, University Goettingen, Goettingen, Lower Saxony, 37077, Germany

^j^ Interdisciplinary Center of Clinical Research (IZKF), Medical Faculty, University of Muenster, Muenster, North Rhine-Westphalia, 48149, Germany

^k^ Lead Contact

^*^ **Corresponding Author:** Stephan Ludwig, Institute of Virology (IVM), Centre for Molecular Biology of Inflammation, University of Muenster, Muenster, North Rhine-Westphalia, Von-Esmarch-Straße 56, 48149, Germany

E-mail: ludwigs@uni-muenster.de

**This file includes:**

**Tables S1 to S3**

**Figures S1 to S6**

**Supplementary Figures and Tables**

**Tab. S1 Reagents and resources**

| Reagent or Resource | Company | Catalog number and identifier |
| --- | --- | --- |
| Antibodies | | |
| rabbit monoclonal anti-SARS-CoV-2 (2019-nCoV) Nucleocapsid (N) | Sino Biologicals | Cat# 40143-R019, RRID:AB_2827973 |
| rabbit polyclonal anti-SARS-CoV-2 spike glycoprotein (S0, S1, S2) | abcam | Cat# ab272504, RRID:AB_2847845 |
| recombinant rabbit monoclonal anti-ACE2 (EPR4435(2)) | abcam | Cat# ab108252  RRID:AB_10864415 |
| rabbit polyclonal anti-CathepsinL | CellSignaling Technology | Cat# 71298 |
| mouse monoclonal anti-phospho-p44/42 MAPK (pERK1/2) (Thr202/Tyr204) (E10) | CellSignaling Technology | Cat# 9106, RRID:AB_331768 |
| rabbit monoclonal anti-p44/42 MAPK (ERK1/2) (137F5) | CellSignaling Technology | Cat# 4695, RRID:AB_390779 |
| rabbit monoclonal anti-phospho-IRF-3 (Ser396) (4D4G) | CellSignaling Technology | Cat# 4947  RRID:AB_823547 |
| recombinant rabbit monoclonal anti-IRF3 (EPR2418Y) | abcam | Cat# ab68481  RRID:AB_11155653 |
| rabbit polyclonal anti-phospho-NFκB p65 (Ser468) | CellSignaling Technology | Cat# 3039  RRID:AB_330579 |
| rabbit polyclonal anti-NFκB p65 (C-20) | SantaCruz | Cat# sc-372  RRID:AB_632037 |
| mouse monoclonal anti-phospho-STAT1 (Tyr701) | BD Biosciences | Cat# 612564, RRID:AB_399855 |
| rabbit polyclonal anti-STAT1 | CellSignaling Technology | Cat# 9172, RRID:AB_2198300 |
| recombinant rabbit monoclonal anti-TMPRSS2 (EPR3861) | abcam | Cat# ab92323  RRID:AB_10585592 |
| mouse monoclonal anti-Tubulin (DM1A) | Sigma-Aldrich | Cat# T6199, RRID:AB_477583 |
| Anti-mouse IgG, HRP-linked Antibody | CellSignaling Technology | Cat# 7076  RIDD:AB_330924 |
| Anti-rabbit IgG, HRP-linked Antibody | CellSignaling Technology | Cat# 7074  RIDD:AB_2099233 |
| Goat IgG Polyclonal anti-rabbit AlexaFluor 568 gamma IgG (H+L) | Invitrogen | Cat# A-11011, RRID:AB_143157 |
| Dapi (4,6-diamidino-2-phenylindole, delictate) | Invitrogen | Cat# D3571, RRID:AB_2307445 |
| Virus Strains | | |
| München-1/02/2020/984  (WT) | München, Germany | N/A |
| hCoV-19/Germany/FI1103201/2020 (D614G-FI) | South Tyrol, Italy | N/A |
| hCoV-19/Germany/LP110320/2020  (D614G-LP) | Ischgl, Austria | N/A |
| hCoV-19/Germany/NK110320/2020  (D614G-NK) | Ischgl, Austria | N/A |
| hCoV-19/Germany/NW-RKI-I-0026/2020 (α-B1.1.7) | Thorsten Wolff, Robert-Koch-Institute | N/A |
| hCoV-19/Germany/NW-RKI-I-0029/2020 (β-B1.351) | Thorsten Wolff, Robert-Koch-Institute | N/A |
| hCoV-19/Germany/202105/2021  (B1.177.81) | Münster, Germany | N/A |
| hCoV-19/Germany/326763/2021  (δ-B1.617.2) | Münster, Germany | N/A |
| Chemicals, Peptides and Recombinant Proteins | | |
| ATR-002 (PD0184264)  2-(2-chloro-4-iodophenylamino)-N-3,4-difluorobenzoic acid | ChemCon GmbH / ATRIVA | N/A |
| Critical Commercial Assays | | |
| CytoSelect^TM^ LDH Cytotoxicity Assay Kit | Cell Biolabs Inc. | Cat# CBA-241 |
| LEGENDplex^TM^ Human anti-virus response panel (13-plex) with V-bottom plate | BioLegend | Cat# 740390 |
| Experimental Models: Cell Lines and Primary Cells | | |
| Human airway epithelial cells (Calu3) | ATCC | Cat# HTB-55, RRID:CVCL_0609 |
| Human alveolar lung epithelial cells (A549) | ATCC | Cat# CRL_7909,  RIDD:CVCL_0023 |
| African green monkey kidney epithelial cells (VeroE6) | Institute of Virology, Muenster (IVM) | N/A |
| Human colorectal adenocarcinoma cells (CaCo2) | Department of Immunology, Tuebingen | N/A |
| Human embryonic kidney cells (HEK293T) | Institute of Virology, Muenster (IVM) | N/A |
| A549-TMPRSS2 | German Primate Center - Leibniz Institute for Primate Research, Goettingen | N/A |
| Vero76-TMPRSS2 | German Primate Center - Leibniz Institute for Primate Research, Goettingen | N/A |
| A549-ACE2 | German Primate Center - Leibniz Institute for Primate Research, Goettingen | N/A |
| A549-ACE2/TMPRSS2 | German Primate Center - Leibniz Institute for Primate Research, Goettingen | N/A |
| AEC (A41.1) (Donor3) | Translational Pediatrics, Department of Pediatrics, University Hospital Wuerzburg | N/A |
| AEC (A43.2) (Donor1) | Translational Pediatrics, Department of Pediatrics, University Hospital Wuerzburg | N/A |
| AEC (A+44.2) (Donor4) | Translational Pediatrics, Department of Pediatrics, University Hospital Wuerzburg | N/A |
| AEC (A+44.3) (Donor2) | Translational Pediatrics, Department of Pediatrics, University Hospital Wuerzburg | N/A |
| AEC (A+45.2) (Donor5) | Translational Pediatrics, Department of Pediatrics, University Hospital Wuerzburg | N/A |
| Oligonucleotides | | |
| SignalSilence® p44/42 MAPK (ERK1/2) siRNA | CellSignaling Technology | Cat# 6560 |
| SignalSilence® Control siRNA (Unconjugated) | CellSignaling Technology | Cat# 6568 |
| Primer: huIFNβ_for:  5´-ggccatgaccaagtgtctcctcc-3´ | This publication | N/A |
| Primer: huIFNβ_rev:  5´-gcgctcagtttcggaggtaacctgt-3´ | This publication | N/A |
| Primer: huMxA_for:  5´-gtttccgaagtggacatcgca-3´ | This publication | N/A |
| Primer: huMxA_rev:  5´-gaagggcaactcctgacagt-3´ | This publication | N/A |
| Primer: huIL-6_for:  5´-agaggcactggcagaaaacaac-3´ | This publication | N/A |
| Primer: huIL-6_rev:  5´-aggcaagtctcctcattgaatcc-3´ | This publication | N/A |
| Primer: huCXCL8_for:  5´-cttgttccactgtgccttggtt-3´ | This publication | N/A |
| Primer: huCXCL8_rev:  5´-gcttccacatgtcctcacaacat-3´ | This publication | N/A |
| Primer: huCXCL10_for:  5´-ggaacctccagtctcagcacca-3´ | This publication | N/A |
| Primer: huCXCL10_rev:  5´-agacatctcttctcacccttc-3´ | This publication | N/A |
| Primer: huCCL2_for:  5´-tcgcctccatcatgaaagtc-3´ | This publication | N/A |
| Primer: huCCL2_rev:  5´-ttgcatctggctgagcgag-3´ | This publication | N/A |
| Primer: huCCL5_for:  5´-cggcacgcctcgctgtcatc-3´ | This publication | N/A |
| Primer: huCCL5_rev:  5´-gcaagcagaaacaggcaaat-3´ | This publication | N/A |
| Primer: GAPDH_for:  5´-gcaaattccatggcaccgt-3´ | This publication | N/A |
| Primer: GAPDH_rev:  5´-gccccacttgatttggagg-3´ | This publication | N/A |
| Software and Algorithms | | |
| GraphPad PRISM version 8.4.3 | GraphPad Software | <http://www.graphpad.com/>  RIDD:SCR_002798 |
| LI-COR Image Studio^TM^ Software version 5.2.5 | LI-COR | https://www.licor.com/bio/  products/software/image_  studio/?gclid=EAIaIQobCh  MIrv7s26ug1wIVQUCGCh  1kvQgLEAAYASAAEgLcY  PD_BwE  RIDD:SCR_015795 |
| AxioVision V4.8.2.0 | Zeiss | http://www.zeiss.com/  microscopy/en_de/products/  microscope-software/  axiovision-for-biology.html  RIDD:SCR_002677 |
| Intensity Ratio Nuclei Cytoplasm Tool (ImageJ macro) |  | https://dev.mri.cnrs.fr  RIDD: N/A |
| LEGENDplex^TM^ Data Analysis Software version 8.0 | BioLegend^®^ | RRID:SCR_001134 |

| **Tab. S2 Primary nasal airway epithelial cell (AEC) isolates** | | | | | | |
| --- | --- | --- | --- | --- | --- | --- |
| **Donor Nr.** | **ID** | **Date** | **Cohort** | **Gender (F/M)** | **Age** |  |
|  |  |  |  |  | **Years** | **Days** |
| 1 | A43.2 | 21/05/2021 | AEC | F | 20 | 7567 |
| 2 | A+44.3 | 21/05/2021 | AEC | M | 84 | 30902 |
| 3 | A41.1 | 24/02/2021 | AEC | F | 22 | 7905 |
| 4 | A+44.2 | 24/02/2021 | AEC | M | 84 | 30902 |
| 5 | A+45.2 | 24/02/2021 | AEC | F | 78 | 28559 |

**Tab. S3 ATR-002 concentrations for cytotoxicity determination**

|  | **1.** | **2.** | **3.** | **4.** | **5.** | **6.** | **7.** | **8.** | **9.** | **10.** | **11.** | **12.** | **13.** | **14.** |
| --- | --- | --- | --- | --- | --- | --- | --- | --- | --- | --- | --- | --- | --- | --- |
| **Conc. [µM]** | **1** | **5** | **10** | **15** | **20** | **40** | **50** | **60** | **80** | **100** | **150** | **200** | **400** | **600** |

| **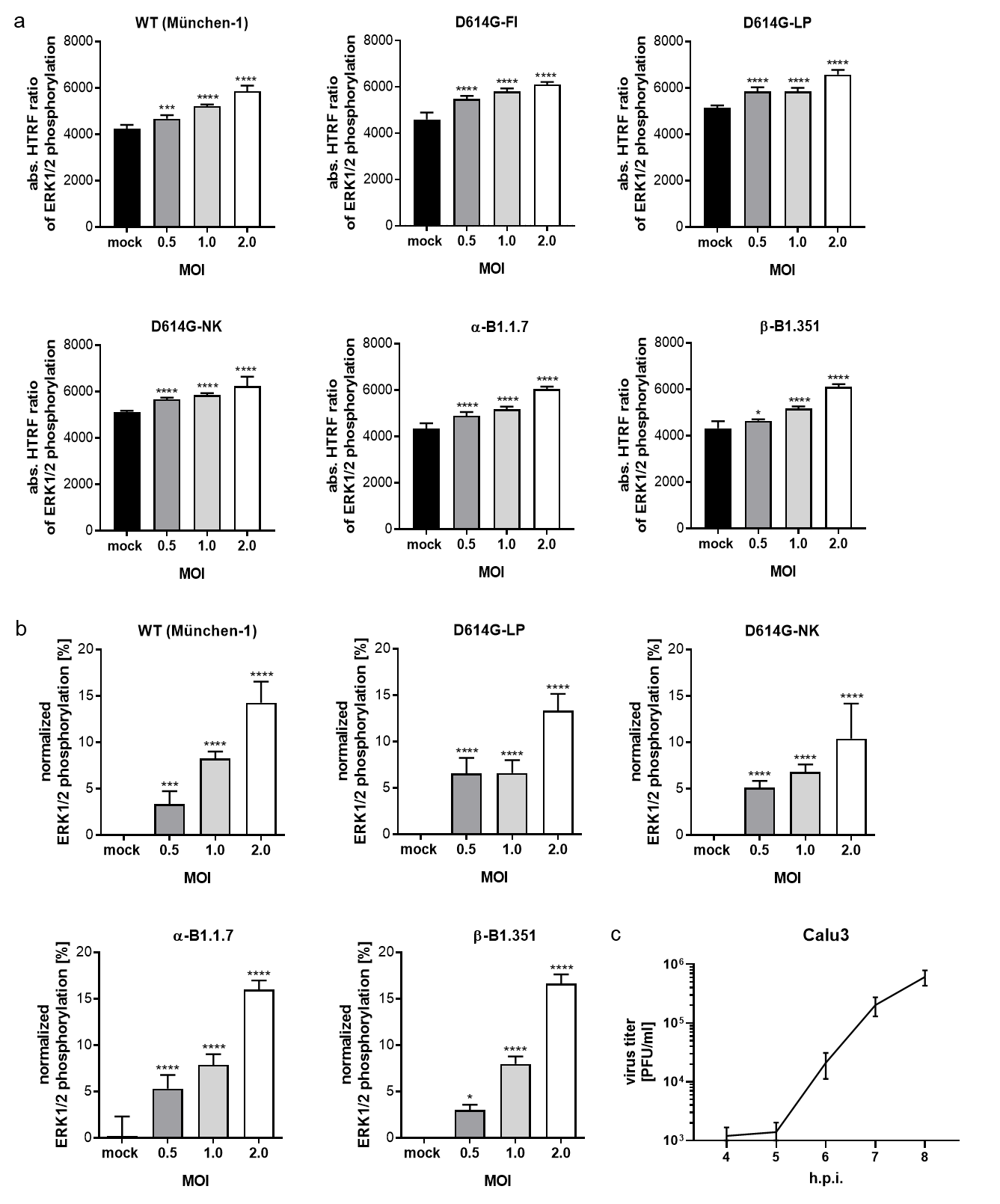** |
| --- |
| **Fig. S1 ERK1/2 activation by SARS-CoV-2 depends on the dose of infection. Related to Fig. 1.**  Calu3 cells were infected with SARS-CoV-2 (Wild type (WT), D614G, α-B1.1.7, β-B1.351) using different MOIs. ERK1/2 phosphorylation was analyzed 1 h.p.i.  **a** Absolute homogenous time resolved fluorescence (HTRF). See also Fig. 1d.  **b** Normalized values of (a). Mock served as reference.  **a, b** Shown are means ± SD of three independent experiments. Data passed an one-way ANOVA followed by Dunnett´s multiple comparison test (* p≤0.0332; *** p≤0.0002; **** p≤0.0001).  **c** Titer analysis of newly produced SARS-CoV-2 virus particles. Supernatants are related to samples of Fig. 1a,b and were analyzed, beginning 4 h.p.i. Shown are results of three independent experiments |

| **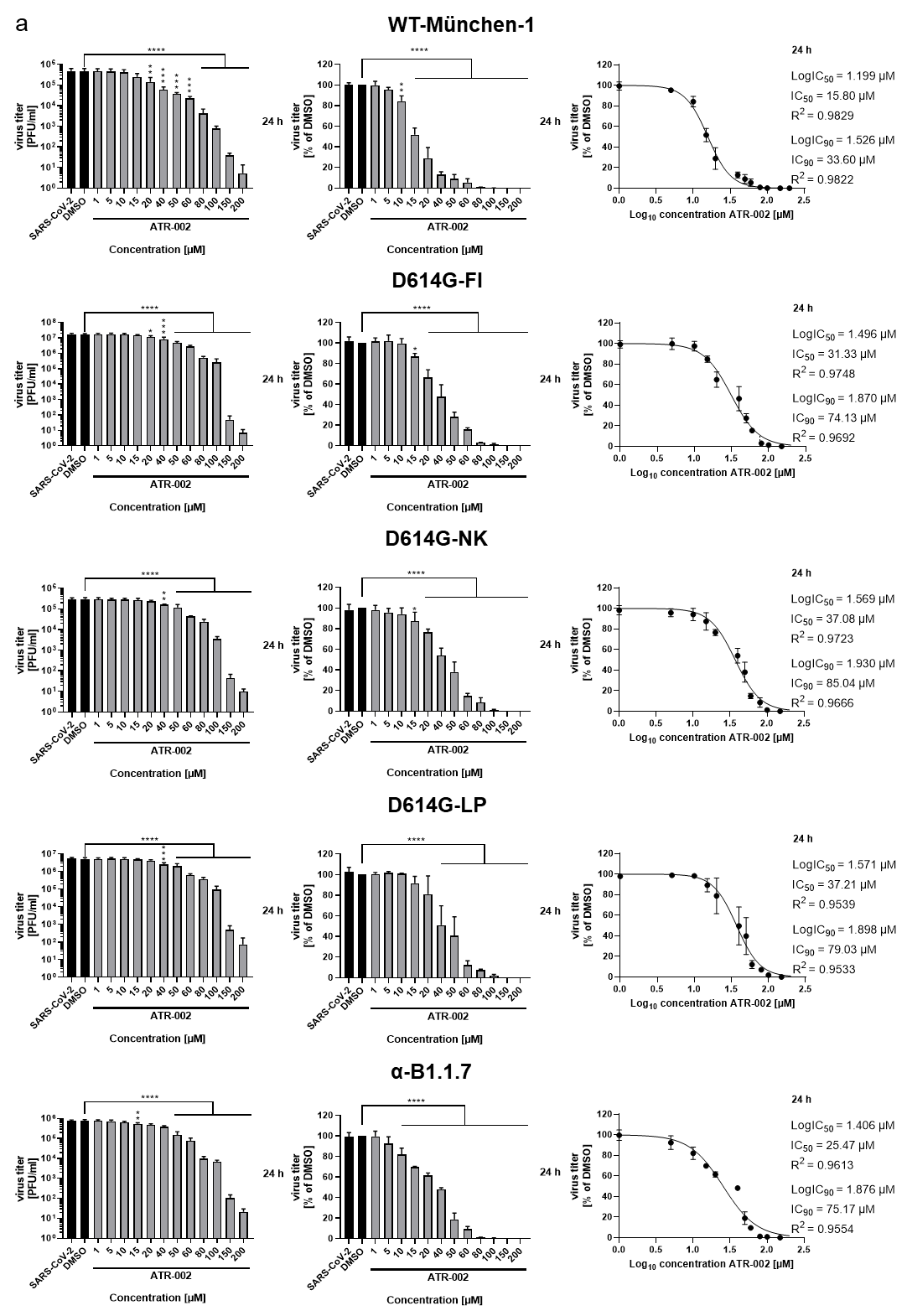** |
| --- |
| **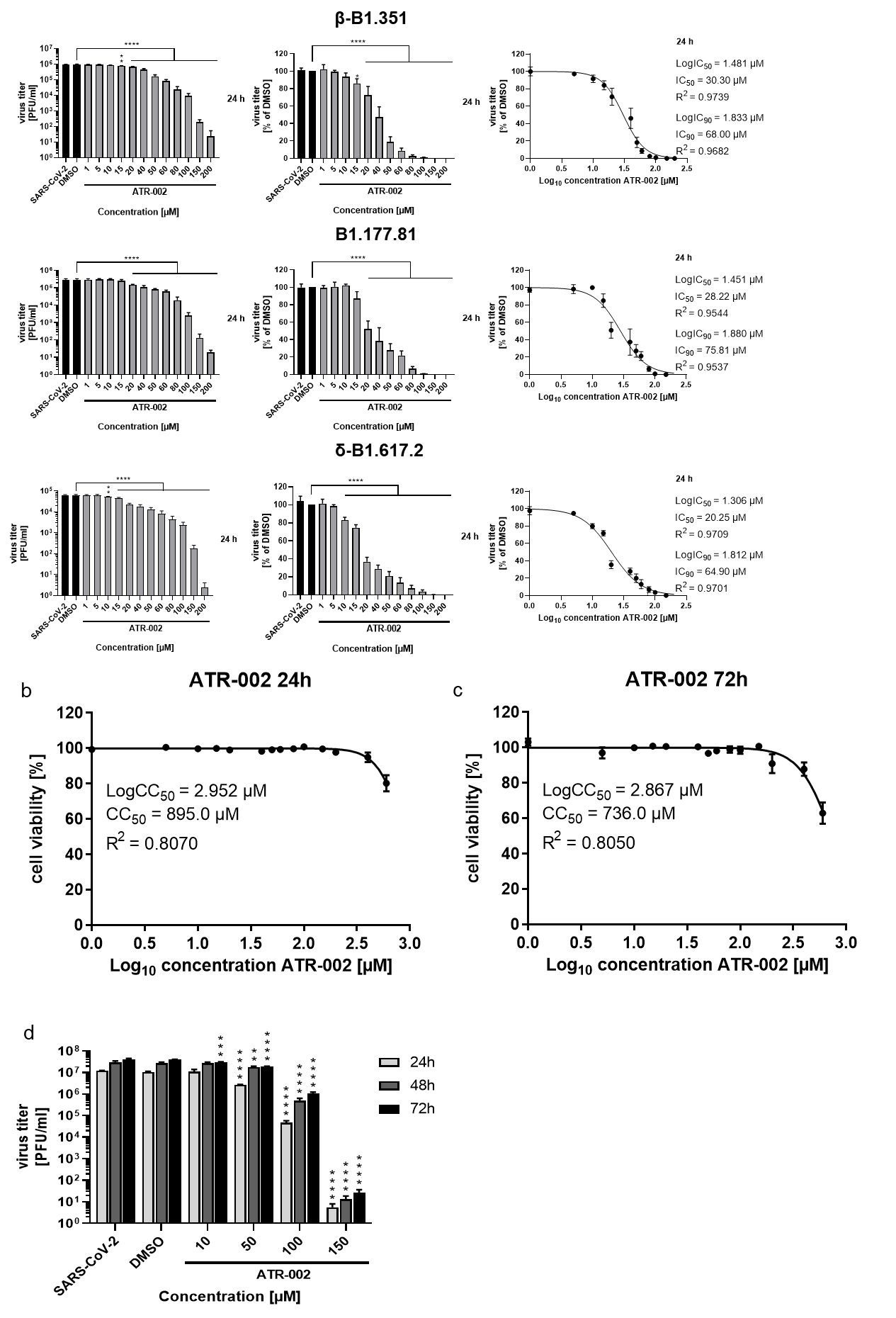**  **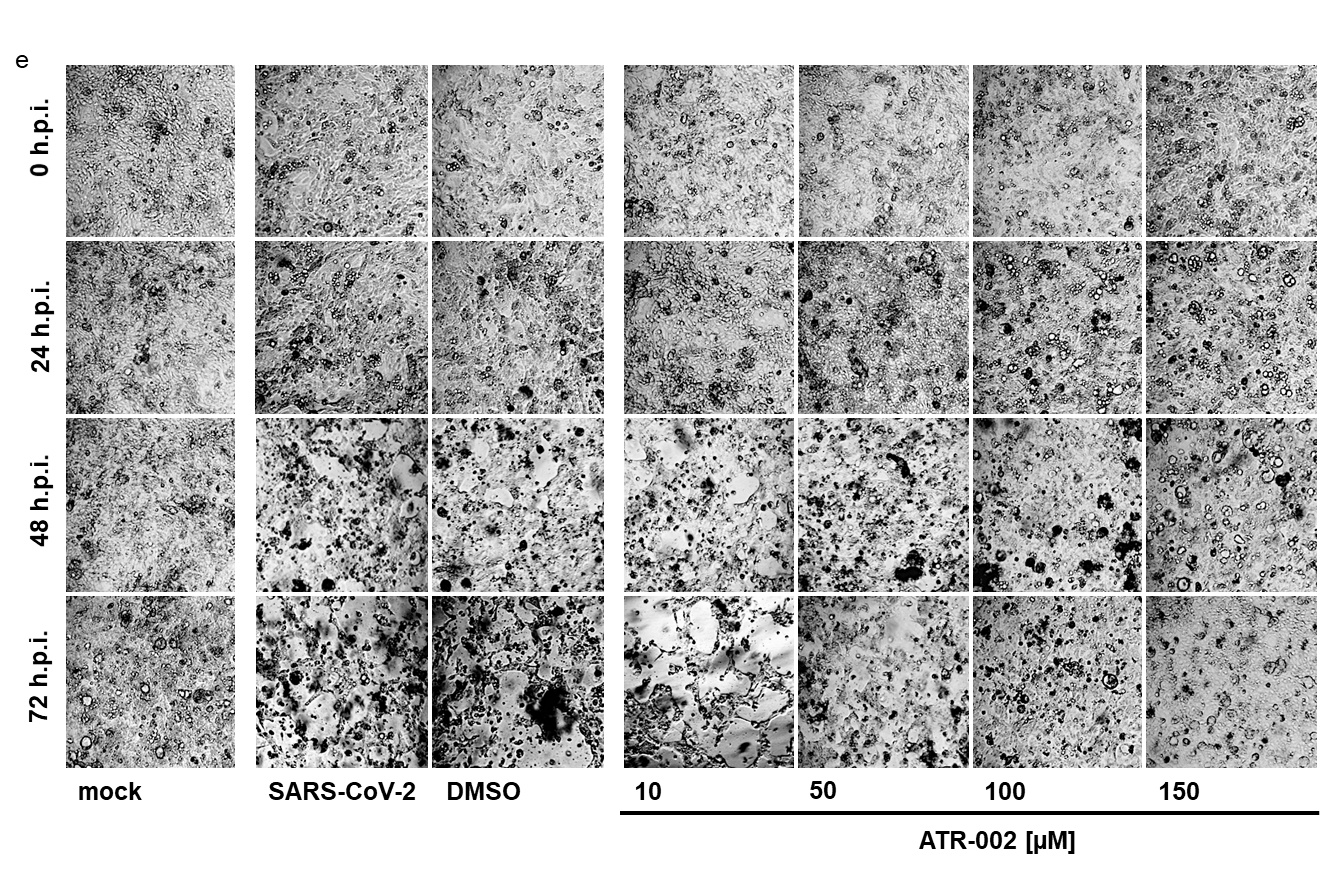**  **Fig. S2 Decreased viral titers of different SARS-CoV-2 variants after ATR-002 treatment. Related to Tab. 1.**  Calu3 cells were infected with different SARS-CoV-2 virus variants (MOI 0.01). 1 h.p.i. cells were treated with ATR-002. SARS-CoV-2 and DMSO served as controls.  **a** Reduction of viral titers in Calu3 cells is dependent on the ATR-002 concentration. See also Tab. 1.  **b, c**  Cytotoxicity evaluation of ATR-002 in Calu3 cells after 24 h and 72 h treatment via LDH assay.  **d** Titer reduction of SARS-CoV-2 in Calu3 cells after ATR-002 treatment. One-way ANOVA was performed for each time point separately.  **e** Light microscopy pictures of one out of three independent experiments of (d).  **a-d** Data represent means ± SD of three independent experiments, each performed in triplicates. Data passed an one-way ANOVA followed by Dunnett´s multiple comparison test (* p≤0.0332; ** p≤0.0021; *** p≤0,0002; **** p≤0.0001). DMSO was used as reference. |

| **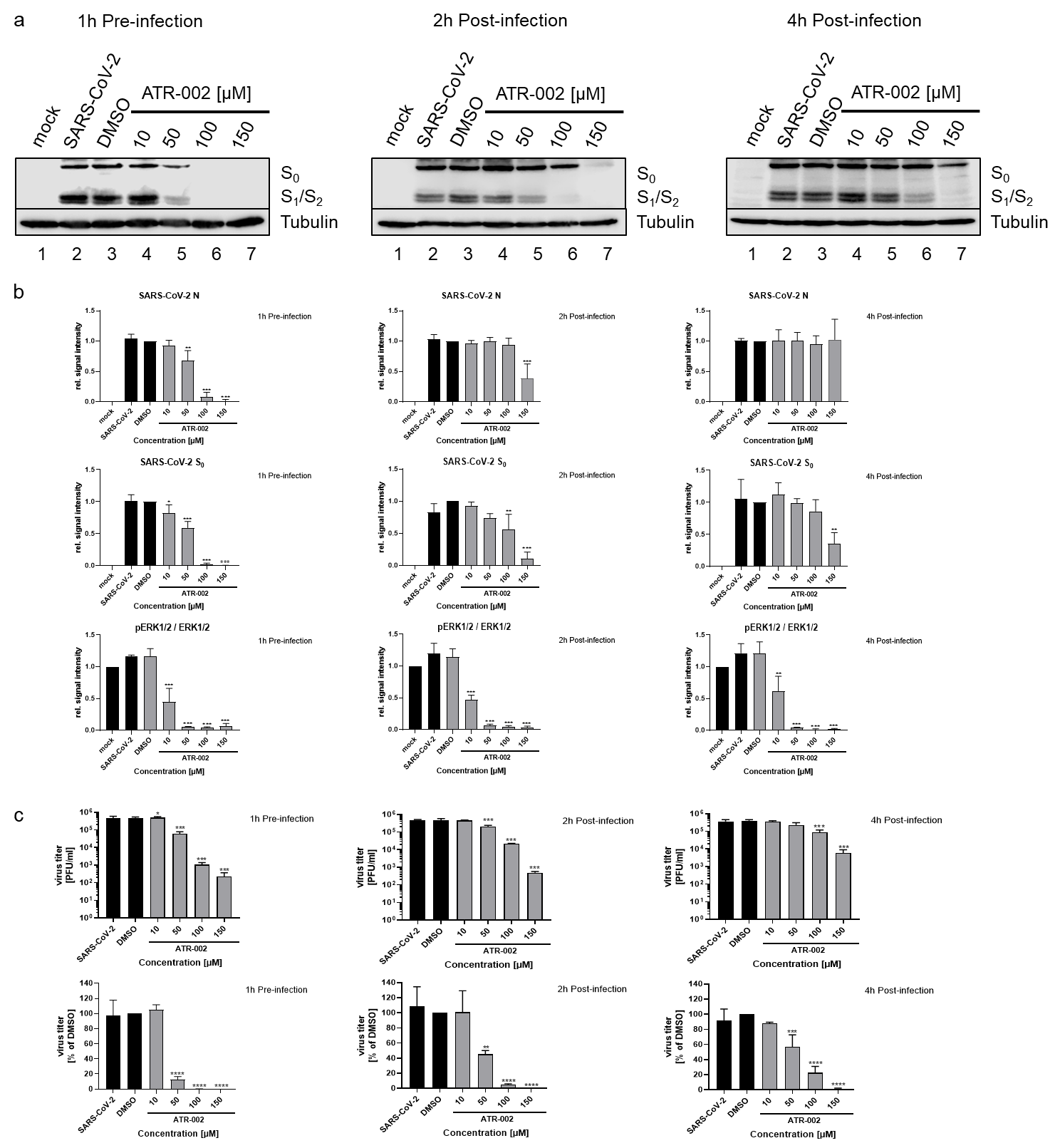** |
| --- |
| **Fig. S3 Timely dependent effect of ATR-002 on the expression of SARS-CoV-2 proteins (N, S) and the release of infectious virus particles. Related to Fig. 3.**  **a** S1/S2 analysis of the results in Fig. 3a. Shown are results of one out of three independent experiments.  **b** Separated analysis of the results in Fig. 3b.  **c** Separated analysis of the titers in Fig. 3c.  **b, c** Data represent means ± SD of three independent experiments. Data passed an one-way ANOVA followed by Dunnett´s multiple comparison test (* p≤0.0332; ** p≤0.0021; *** p≤0.0002; **** p≤0.0001). DMSO was used as reference. |

| **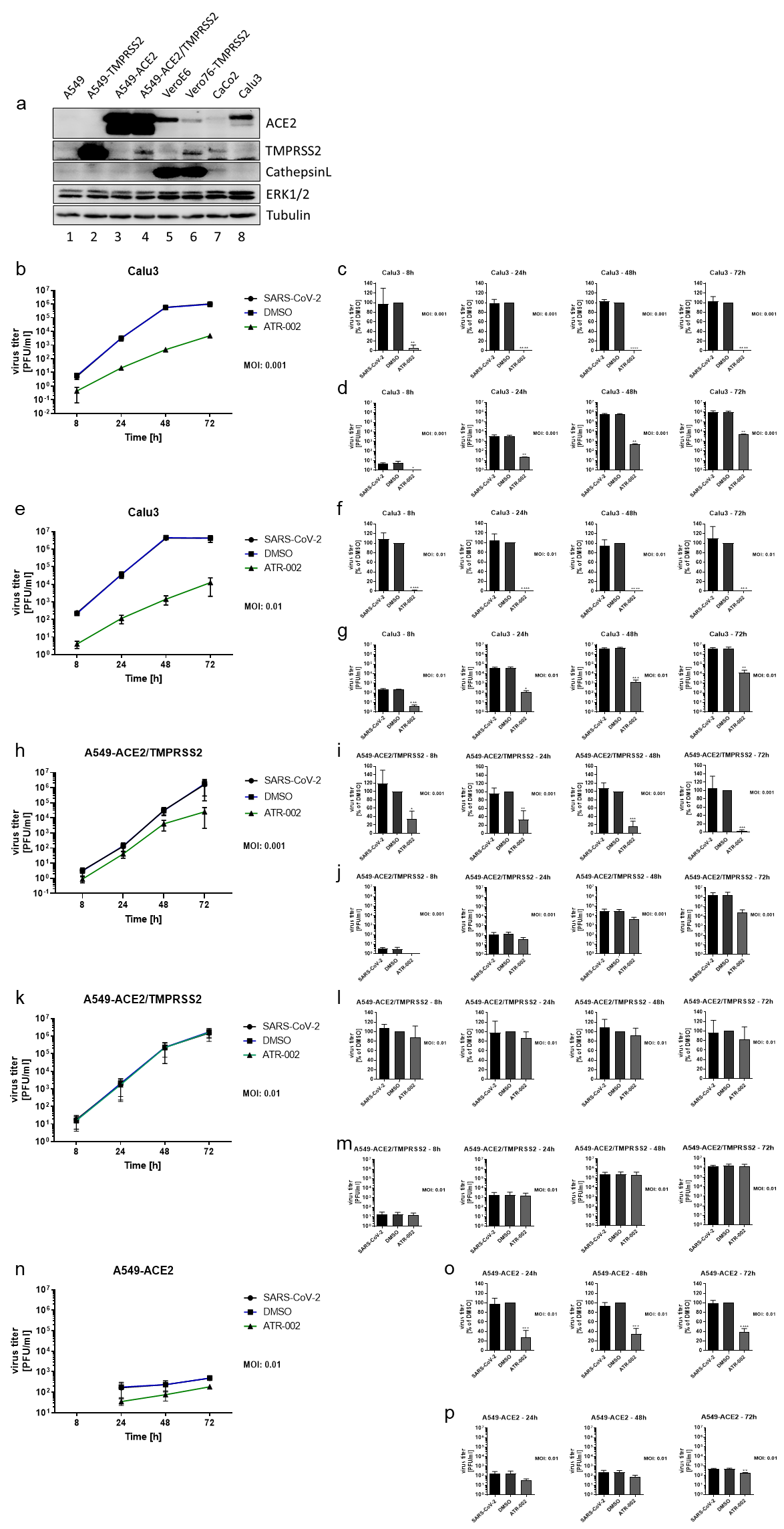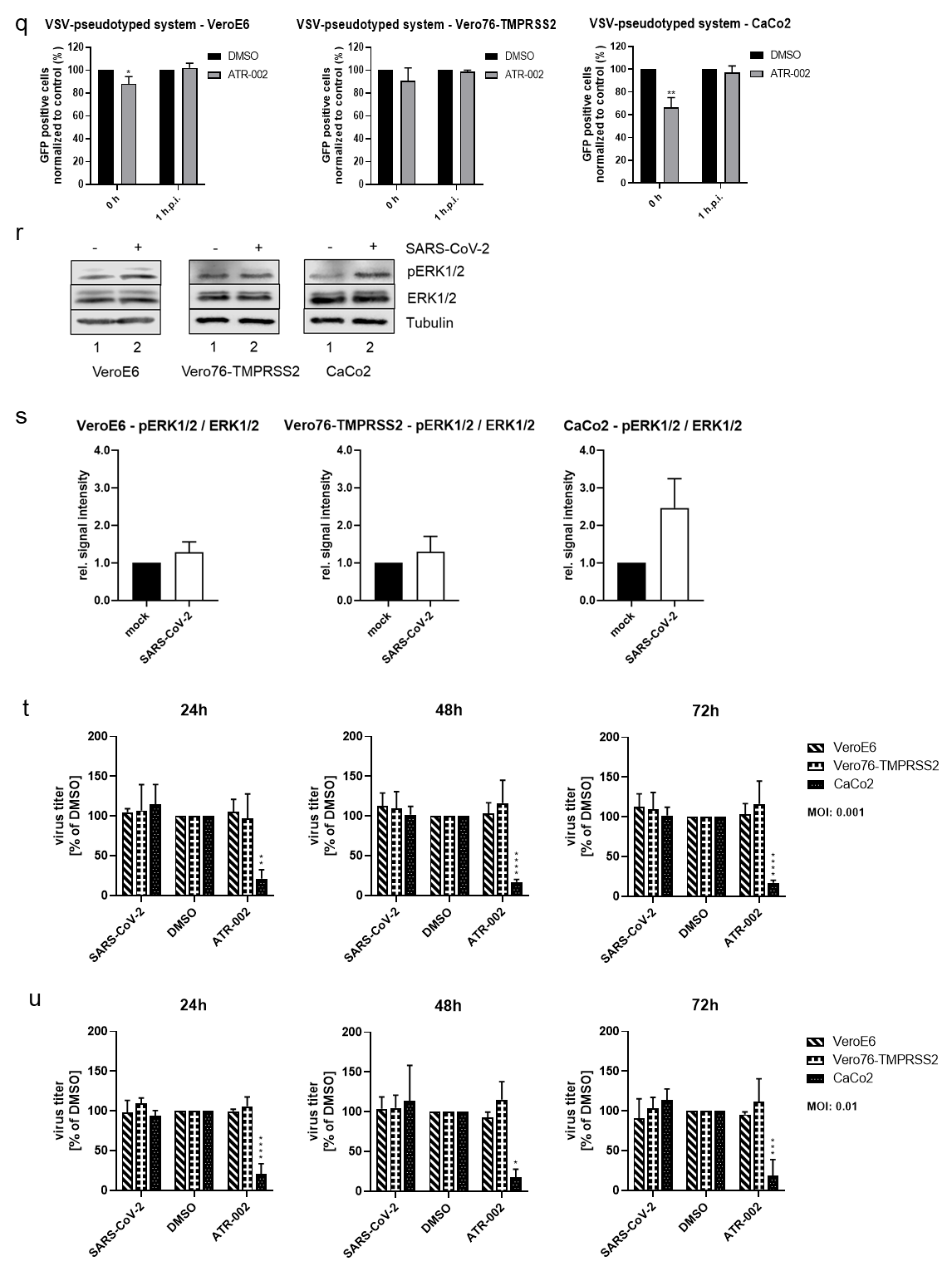** |
| --- |
| **Fig. S4 MEK1/2-inhibition in Calu3, A549-ACE2 and CaCo2 cells results in decreased viral titers. Related to Fig. 4.**  **a** Expression analysis of ACE2, TMPRSS2 and Cathepsin L in the different cell lines. Immunoblots were probed with anti-ACE2, anti-TMPRSS2, anti-Cathepsin L, anti-ERK1/2 and anti-Tubulin antibodies.  **b-p, t, u** Cells were infected with SARS-CoV-2 (D614G-FI) using different MOIs. Untreated (SARS-CoV-2) and DMSO treated cells served as controls.  **b, e, h, k, n** Growth kinetic of SARS-CoV-2 (D614G-FI) using different MOIs (b,h: 0.001; e,k,n: 0.01). Data represent means ± SD of three independent experiments, each performed in triplicates. See also Fig. 4b.  **c, d, f, g, i, j, l, m, o, p** 1 h.p.i. cells were treated with ATR-002 (100 µM). Untreated (SARS-CoV-2) and DMSO (0.1 %) treated cells served as negative controls. Data passed an one-way ANOVA followed by Dunnett´s multiple comparison test (* p≤0.0332; ** p≤0.0021; *** p≤0,0002; **** p≤0.0001). DMSO was used as reference and was for percentage calculation arbitrarily set to 100 %. See also Fig. 4e.  **q** VeroE6, Vero76-TMPRSS2 and CaCo2 cells were infected with the VSV-pseudotyped system VSV ΔG/GFP-Luc + S- Δ21. 0 h or 1 h.p.i. ATR-002 (100 µM) treatment was initiated. Infected cells were incubated for 16 h in the presence of ATR-002.  **r** Cell lines were infected with SARS-CoV-2 (FI) (MOI 2) (+).1 h.p.i. immunoblots were prepared and anti-pERK1/2, anti-ERK1/2 and anti-Tubulin antibodies were used to analyze the phosphorylation state. Shown are results of one out of three independent experiments. Mock (-) infected cells served as negative control.  **s** Quantification of (r). pERK1/2 intensity ratios were normalized to the total protein amount. Mock was arbitrarily set to 1.0. Data passed a paired two-tailed *t*-test (ns) for each cell line individually.  **t, u** Titer reduction of SARS-CoV-2 (FI) in VeroE6, Vero76-TMPRSS2 and CaCo2 cell lines after ATR-002 (100 µM) treatment. 1 h.p.i. ATR-002 treatment was initialized. Untreated (SARS-CoV-2) and DMSO (0.1 %) treated cells served as negative controls. Data passed an one-way ANOVA followed by Dunnett´s multiple comparison test (* p≤0.0332; ** p≤0.0021; *** p≤0.0002; **** p≤0.0001) for each cell line separately. DMSO was used as reference and arbitrarily set to 100 %.  **b-q, s-u** Data represent means ± SD of three independent experiments, each performed in triplicates (b-p, s-u) or in quadruplicates (q). |

| **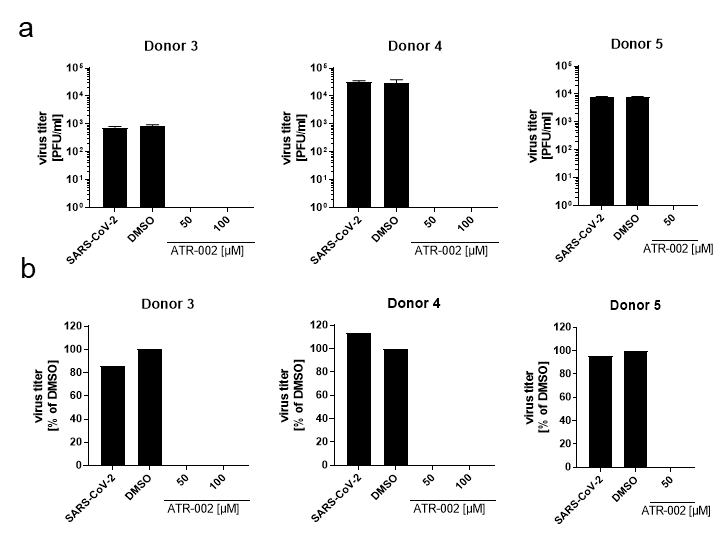** |
| --- |
| **Fig. S5 SARS-CoV-2 replication is efficiently blocked in air-liquid-interface cultures by ATR-002. Related to Fig. 5.**  SARS-CoV-2 (D614G-FI) (MOI 1) was used to apically infect primary nasal airway epithelial cells (AEC). Basolateral ATR-002 (50 µM, 100 µM) treatment was initiated 2 h.p.i. for 48 h followed by an apical incubation with 200 µl culture medium for 20 min. Untreated (SARS-CoV-2) and DMSO (0.1 %) treated cells served as negative controls. Data shows results of one experiment.  **a** Virus titer in PFU/ml.  **b** Virus titer in percentage. DMSO was arbitrarily set to 100 %. |

| 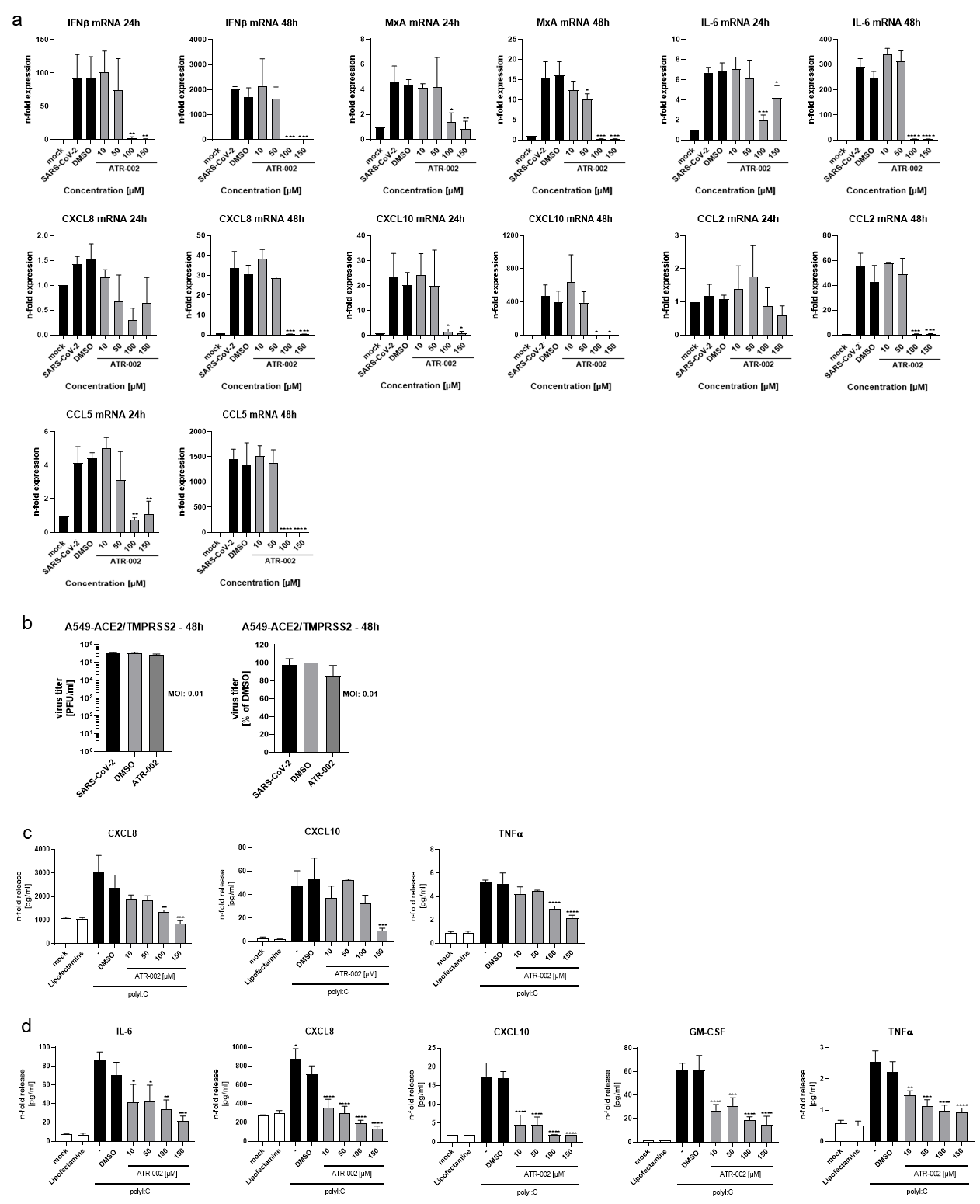 |
| --- |
| **Fig. S6 Reduced cytokine expression after ATR-002 treatment. Related to Fig. 6.**  **a** Separated analysis of mRNA expression shown in Fig. 6a. Calu3 cells were infected with SARS-CoV‑2 (D614G-FI) (MOI 0.01). 1 h.p.i. cells were treated with ATR-002. Mock, SARS-CoV-2 and DMSO served as controls.  **b** Titer analysis of Fig. 6b  **c, d** Absolute values of the cytokine release in Calu3 (c) and A549-ACE2/TMPRSS2 (d) analysis shown in Fig. 6d, e.  **a-d** Data passed an one-way ANOVA test followed by Dunnett´s multiple comparison test (* p≤0.0332; ** p≤0.0021; *** p≤0,0002; **** p≤0.0001). DMSO was used as reference. |
